# Early alteration of epigenetic-related transcription in Huntington’s disease mouse models

**DOI:** 10.1038/s41598-018-28185-4

**Published:** 2018-07-02

**Authors:** Irati Hervás-Corpión, Deisy Guiretti, Manuel Alcaraz-Iborra, Román Olivares, Antonio Campos-Caro, Ángel Barco, Luis M. Valor

**Affiliations:** 1 Unidad de Investigación, Hospital Universitario Puerta del Mar, Instituto de Investigación e Innovación en Ciencias Biomédicas de Cádiz (INiBICA), Av. Ana de Viya 21, 11009 Cádiz, Spain; 2 Instituto de Neurociencias, Universidad Miguel Hernández – Consejo Superior de Investigaciones Científicas, Av. Santiago Ramón y Cajal s/n, Sant Joan d’Alacant, 03550 Alicante, Spain; 3 Present Address: Instituto de Histología y Embriología (IHEM, CONICET/UNCuyo), Facultad de Ciencias Médicas, CC56, Universidad Nacional de Cuyo, 5500 Mendoza, Argentina; 4 Present Address: Departamento de Neurociencia y Ciencias de la Salud, Universidad de Almería, Carretera Sacramento s/n, La Cañada de San Urbano, 04120 Almería, Spain

## Abstract

Transcriptional dysregulation in Huntington’s disease (HD) affects the expression of genes involved in survival and neuronal functions throughout the progression of the pathology. In recent years, extensive research has focused on epigenetic and chromatin-modifying factors as a causative explanation for such dysregulation, offering attractive targets for pharmacological therapies. In this work, we extensively examined the gene expression profiles in the cortex, striatum, hippocampus and cerebellum of juvenile R6/1 and N171-82Q mice, models of rapidly progressive HD, to retrieve the early transcriptional signatures associated with this pathology. These profiles were largely consistent across HD datasets, contained tissular and neuronal-specific genes and showed significant correspondence with the transcriptional changes in mouse strains deficient for epigenetic regulatory genes. The most prominent cases were the conditional knockout of the lysine acetyltransferase CBP in post-mitotic forebrain neurons, the double knockout of the histone methyltransferases Ezh1 and Ezh2, components of the polycomb repressor complex 2 (PRC2), and the conditional mutants of the histone methyltransferases G9a (Ehmt2) and GLP (Ehmt1). Based on these observations, we propose that the neuronal epigenetic status is compromised in the prodromal stages of HD, leading to an altered transcriptional programme that is prominently involved in neuronal identity.

## Introduction

Huntington’s disease (HD, OMIM# 143100) is the most prevalent polyglutamine (polyQ) disorder, with 5–10 cases per 100,000 inhabitants worldwide. It is caused by an aberrant expansion of CAG repeats in exon 1 of the Huntingtin gene and comprises a complex symptomatology of involuntary movements, mood changes and cognitive deficits^1^, with an age of onset of approximately 30–50 years in the classical variant of the disease and below 20 years in the juvenile variant (affecting 5–10% of patients)^2^. Although medium spiny neurons in the striatum are especially sensitive to the actions of polyQ expansion, other cell types and brain areas can also be affected during the disease progression^3–6^. Notably, the cerebellum is marginally affected in middle-aged adults but can make an important contribution in juvenile cases^7,8^.

Brain malfunction and neurodegeneration in HD are provoked by the disruption of a variety of cellular processes, including transcriptional regulation. Since the first genome-wide survey of the transcriptional changes associated with HD^9^, growing evidence has indicated that this dysregulation is an extensive and early event that is not restricted to the nervous system in animal and cellular models but is also documented in peripheral blood and post-mortem brains from patients^10,11^. Far from being exhausted, the transcriptomics approach continues to provide novel insights regarding the implication of the altered patterns of gene expression in HD. To mention a few examples, recent work in mouse models has identified the molecular networks more tightly correlated with the number of CAG repeats and age^12^, has demonstrated the influence of the genetic background in the magnitude and age of onset of the transcriptional dysregulation^13^ and has revealed the dysregulation of enhancer RNAs, a class of non-coding RNAs transcribed from active enhancers^14^. In humans, transcriptomics studies have been conducted to understand the role of myeloid cells in the inflammatory processes in HD^15^, to identify the widespread RNA splicing dysregulation in the motor cortex of patients^16^ and to propose common aetiopathological mechanisms for several neurodegenerative disorders^17^.

Attempts to explain the perturbed transcriptional programme in HD pinpoint the involvement of multiple transcription factors, chromatin-associated proteins and non-coding RNAs^10,11^. Epigenetic dysregulation has offered an attractive hypothesis to explain the coordinated disruption of multiple genes and the reversion of certain molecular and phenotypic traits after treatment with chromatin-modifying drugs, proven to be beneficial at the preclinical stage^18–20^. Starting with the colocalization of the lysine acetyltransferase CREB-binding protein (CBP) in huntingtin inclusions^21^ and the aberrant subcellular distribution pattern of histone deacetylase interaction partners^22^, increasing evidence has accumulated in recent years by enlarging the catalogue of DNA and histone covalent modifications that are affected in animal and cellular models^19,20^, and extended to patients’ brains^23–25^. Up to now, high-throughput resolution studies revealed that epigenetic dysregulation in HD, despite not fully coinciding with the altered patterns of gene expression, affects relevant genes for neuronal functioning and identity^23,26–29^.

Here, we updated the definition of transcriptional disease signatures in the early stages of the disease. More precisely, we investigated the transcriptomes of different brain structures from juvenile mice of the rapidly progressive R6/1 and N171-82Q strains, which are suitable animal models for juvenile HD. In this manner, we were able to obtain a complete picture of the premature transcriptional effects of mHtt expression in the brains of models for the early onset variant of HD for which information is still scarce. Using an unbiased approach, we observed a significant resemblance of the resulting signatures with the *in vivo* transcriptional consequences of mutations in different epigenetic activities.

## Results

### Defining an early neuronal transcriptional signature for rapidly progressive Huntington’s disease

**Figure 1:**
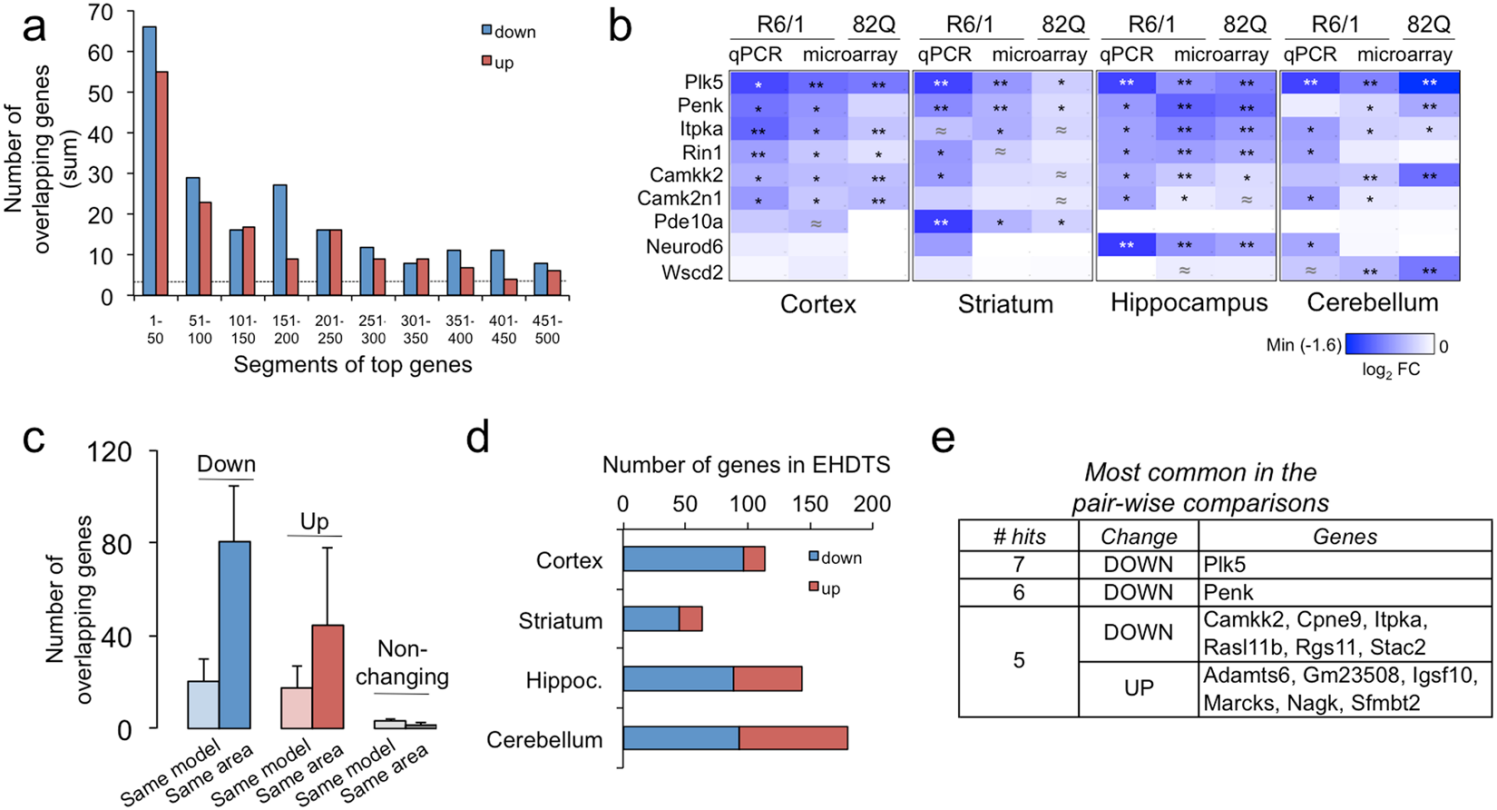
mHtt expression produces early and distinctive transcriptional signatures across brain areas. (**a**) Sum of the number of overlapping genes in any of the eight pair-wise comparisons between genotypes. The results of each comparison were ranked according to the *t* statistic and divided in bins of 50 genes. Bins belonging to the same rank were compared in all the possible pairs. The dashed line represents the random number of overlapping genes. (**b**) RT-qPCR assays for selected genes in independent 7-week-old samples: n = 6 (wt), n = 10 (R6/1). As the microarrays were hybridized from male samples, RNA was extracted from female animals to ensure that the gene expression changes were gender-independent. The colour palette depicts the log_2_-fold change compared to the wild-type condition. Microarray results are shown for comparison purposes. ≈*P* < 0.1; *P < 0.05; **P < 0.001 (Student’s t-test for qPCR, moderated t-test for microarrays). (**c**) Mean ± s.d. of the number of overlapping Top-down, Top-up or non-changing genes (i.e., showing a *P*-value close to 1) between any two areas within a mouse model (*Same model*) or between both models for each brain area (*Same area*). (**d)** The corresponding number of EHDTS for each brain area. (**e)** Genes appearing most frequently in the Top-down and Top-up early signatures in all the pair-wise comparisons HD *vs* wt. # hits, number of pair-wise comparisons in which the genes overlapped.

To elucidate the gene expression changes in early stages of HD, we conducted a microarray analysis in the prefrontal cortex, the striatum, the hippocampus and the cerebellum of N171-82Q and R6/1 mice. The sampling was made in juvenile individuals (ranging between 6 and 7.5 weeks old), after full completion of postnatal development^30^ and preceding the onset of detectable pathological phenotypes^26,31^. The differential expression analysis was conducted by comparing transgenic with wild-type littermates in each brain area and model per separate, and considered statistical subthreshold changes (i.e., nominal *P*-value < 0.05 but adjusted *P*-value > 0.05) to include genes known to be affected in HD. Thus, we assumed that minor alterations at these early stages could be of relevance during disease progression. Once the genes were ranked according to statistical significance, we selected the top 250 downregulated and upregulated genes for downstream analyses (Top-down and Top-up respectively, Supplementary Table 1), as this number was sufficient to contain the common deregulated genes (DRG) between animal models and brain areas (Fig. 1a). In general terms, qPCR assays in independent samples confirmed the transcriptional changes in selected genes (Fig. 1b).

All the possible comparisons between Top-down and Top-up DRGs revealed a high degree of overlap between the same brain areas (Fig. 1c). Thus, we defined the early HD transcriptional signatures (EHDTS) as the common genes between both models in each brain area: 114 DRGs in the prefrontal cortex, 63 DRGs in the striatum, 143 DRGs in the hippocampus, and 180 DRGs in the cerebellum (Fig. 1d and Supplementary Table S1). The lowest number of overlapping genes was observed in the striatum due to the low expression of the mHtt transgene in our N171-82Q colony^26^ that might explain the absence of DRGs in all the eight pair-wise comparisons (Fig. 1e). Despite this limitation, the comparison with publicly available gene profiles from ArrayExpress and GEO repositories confirmed the presence of the EHDTS among the most downregulated genes in the striatum of other HD animal models (see Materials and Methods, Supplementary Fig. S1a and b). In contrast, no significant association was found in either the striatum or the cerebellum of models showing Parkinson’s disease (PD)-related motor impairment (Supplementary Fig. S1b), suggesting that the EHDTS were disease-specific. However, some HD datasets that represented early disease stages in slow progressive models did not show a significant proportion of the EHDTS. To clarify the longitudinal behaviour of our signatures in consistent datasets, we used the RNA-seq data from the most compelling study to date in knock-in (KI) models^12^. In these profiles, we detected a significant presence of the EHDTS in most DRGs in the KI striatum, which positively correlated with the number of CAG repeats and the age at sampling (Supplementary Fig. S1c), demonstrating the tight relationship of the signature with the pathology progression. This presence was observed in animals developing the first signs of motor impairment before an overt pathological behaviour^32,33^, indicating that the striatal EHDTS contained markers related to prodromal stages.

**Figure 2:**
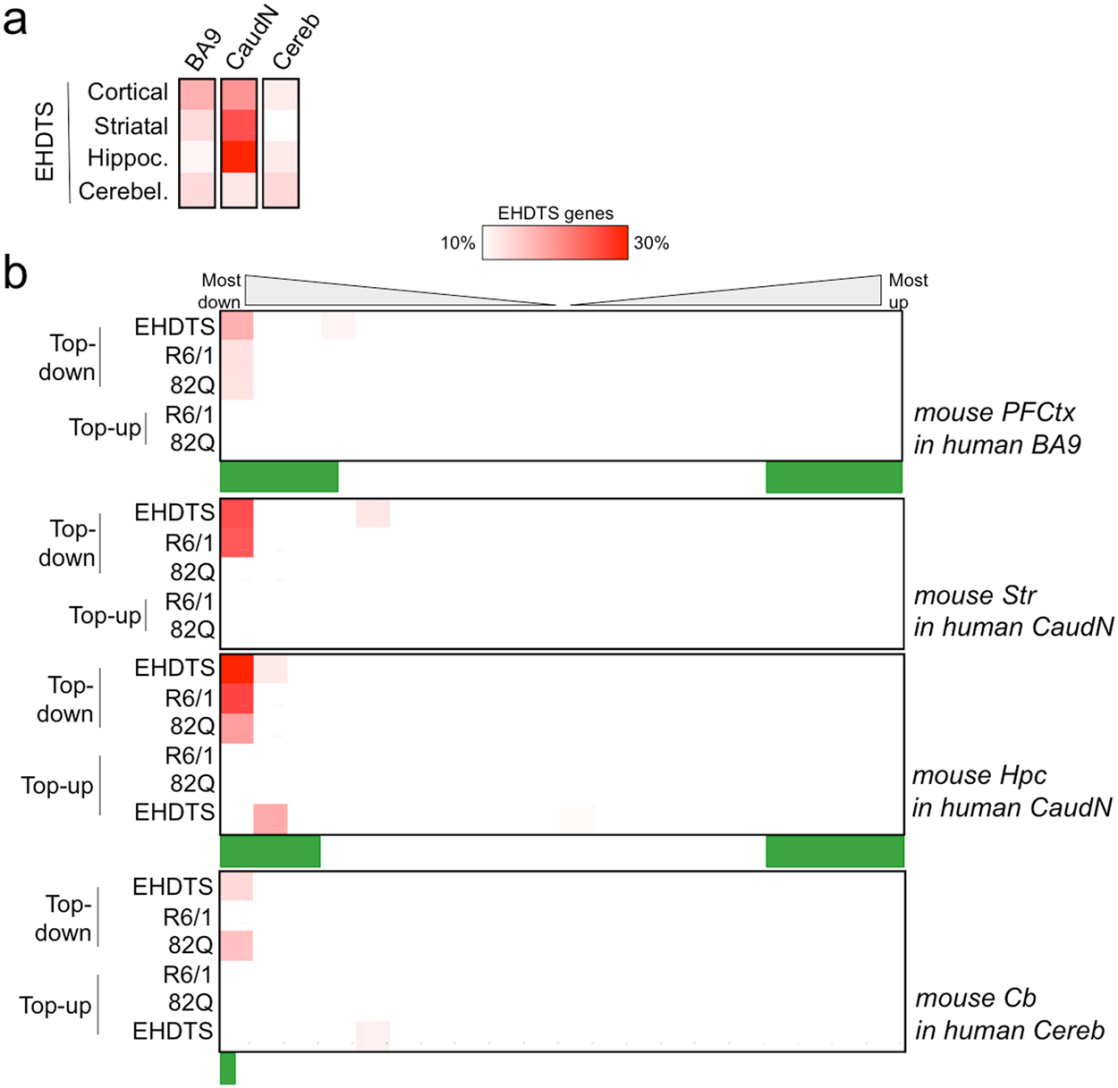
The early transcriptional signatures in mHtt-expressing brains are altered in human patients. (**a**) Percentage of tissue-related EHDTS overlapping with the most downregulated genes in different brain areas from HD patients: the cortical Brodmann Area 9 (BA9), the caudate nucleus (CaudN) and the cerebellum (Cereb). (**b**) The same as in (**a**), extended to the whole human transcriptome, ranked according to *t* statistic and divided into 20 bins of equal numbers of genes (771). In other words, (**a)** depicts the first bin. We also counted the number of Top-down or Top-up genes in each mouse model (R6/1 and 82Q). Green box, region of significant changes according to the original publications (*P* < 0.0001^35^; adj. *P* < 0.05^34^). In contrast to downregulation, upregulated genes in HD mice failed to deviate from a random distribution across the human transcriptomes.

Importantly, most genes contained in the EHDTS were also altered in the transcriptomes derived from post-mortem brains of HD patients^34,35^ (Fig. 2a). As expected, the largest fractions of EHDTS from the prefrontal cortex, the striatum and the cerebellum were found among the top downregulated genes in the human Brodmann area 9 (BA9), the caudate nucleus and the cerebellum, respectively. In the case of the hippocampus, which was absent in the transcriptional studies of HD patients, the highest overlap was identified within the caudate nucleus profile (Fig. 2a); therefore, we used this transcriptome as reference to examine the distribution of the mouse hippocampal alterations across human gene profile (Fig. 2b). Approximately 24–30% of the downregulated EHDTS accumulated in the most downregulated part of the differential transcriptome of the caudate nuclei: *P* < 0.00001, χ^2^ = 133.6, d.f. = 19 for the striatum; *P* < 0.00001, χ^2^ = 160.1, d.f. = 19 for the hippocampus. Less pronounced was the presence of the cortical EHDTS among the most downregulated genes in BA9 (*P* = 4.5 × 10^−5^, χ^2^ = 53.1, d.f. = 19). For its part, the cerebellar signature was found in the top downregulated genes of the corresponding human brain area (Fig. 2b) but did not reach significance (*P* = 0.105, χ^2^ = 27, d.f. = 19), probably because the cerebellum is much less affected in adult patients compared to the basal ganglia^35^. We also mapped the Top-down and Top-up genes from the N171-82Q and R6/1 brains, and we observed that the profile of the R6/1 model was more related to the altered patterns of the human caudate nuclei compared to the N171-82Q model (*P* < 0.00001, χ^2^ = 79.7, d.f. = 19 for R6/1, *P* = 0.483, χ^2^ = 18.6, d.f. = 19 for N171-82Q), in agreement with the different mHtt expression in the striatum between both models^26,36^. Common genes between early mouse and human DRGs are shown in Supplementary Table S3.

### The early transcriptional signatures contain tissular and neuronal-enriched markers

**Figure 3:**
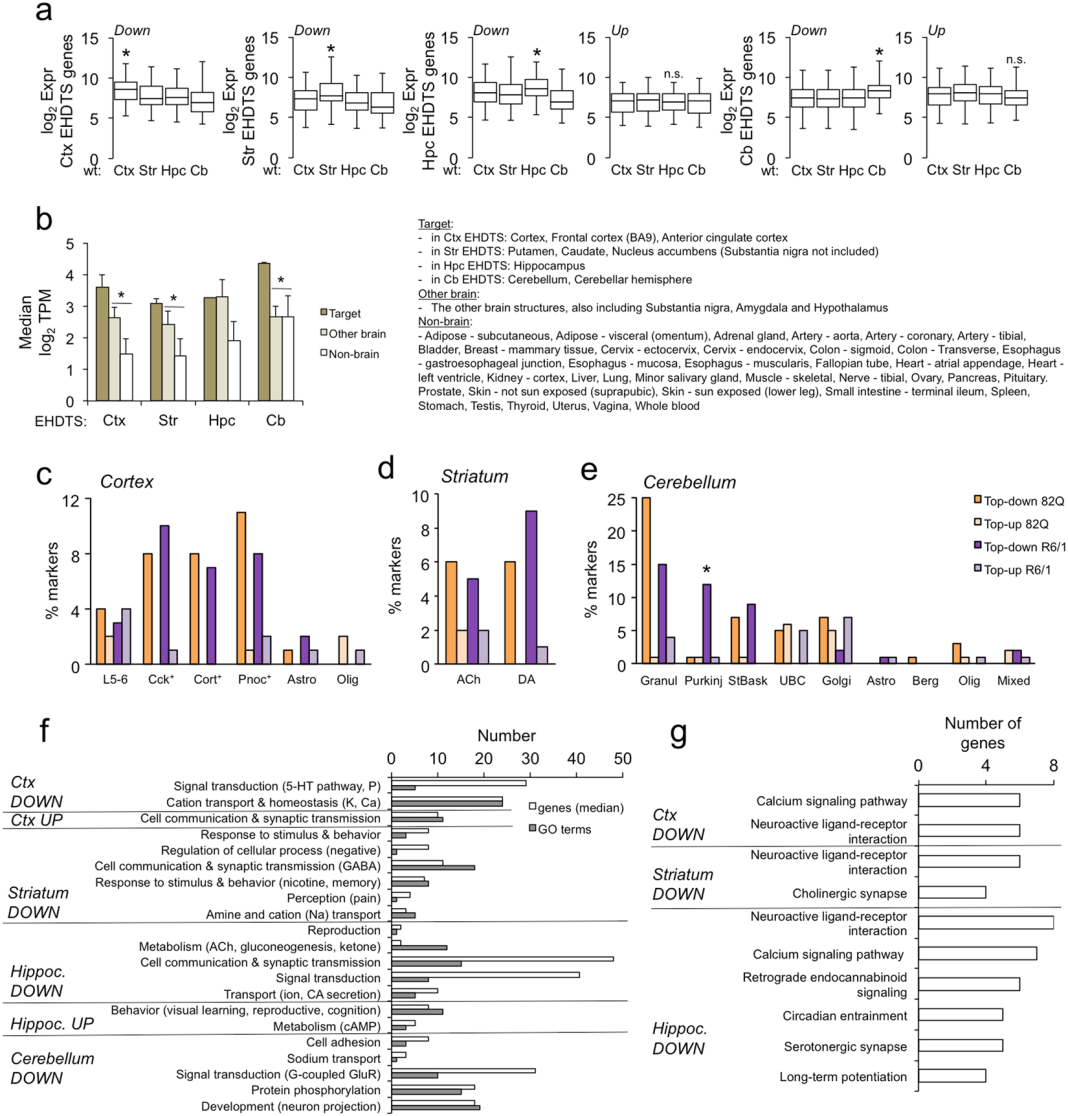
The early transcriptional signatures contain a prominent tissue and neuron-enriched component. (**a)** Microarray expression levels (in log_2_ arbitrary units) of the EHDTS defined for each brain area in wild-type mice. The numbers of upregulated genes in the cortex and the striatum were too low to be plotted. *P < 0.05 in all the comparisons between the values in the tissue of interest and the values of another tissue (Student’s t-test). (**b)** Median of transcripts per million (TPM) for the genes belonging to EHDTS across brain and non-brain tissues from human donors, as deposited in the GTEx portal. Data are expressed as the mean ± s.d. *P < 0.05 between the corresponding (Target) and the remaining brain tissues (Other brain) (Student’s t-test); the comparison with non-brain tissues was also significant. (**c–e**) Percentage of cell-enriched TRAP markers also present in the Top-down and Top-up genes in each HD mouse model in the cortex (**c**), the striatum (**d**), and the cerebellum (**e**). L5-6, neurons from layers 5 and 6; Cck^+^, cholecystokinin-expressing neurons; Cort^+^, corticosterone-expressing neurons; Pnoc^+^, prepronociceptin-expressing neurons; Astro, astrocytes; Olig, oligodendrocytes; Ach, cholinergic neurons; DA, dopaminergic (Drd1, Drd2) neurons; Granul, granule cells; Purkinj, Purkinje cells; StBask, stellate and basket cells; UBC, unipolar brush cells; Golgi, Golgi cells; Berg, Bergmann glia; Mixed, mixed oligodendrocytes (precursors). *P < 0.05, R6/1 *vs* N171-82Q (Fisher’s exact test). (**f**,**g)** Enrichment analysis of Gene Ontology (GO) (**f**) and KEGG pathways (**g**) of the EHDTS. The significant GO terms were manually grouped into the categories shown in the graph. The number of GO terms and the genes therein for each category are depicted. 5-HT, serotonin; P, phosphorylation; CA, catecholamine; cAMP, cyclic adenosine monophosphate; GluR, glutamate receptor. No KEGG pathway was enriched in cerebellum.

The results of Fig. 1c suggested that the transcriptional downregulation observed at early stages of rapidly progressive HD models contained a strong tissue-specific component, which was confirmed in the comparison of the EHDTS with the top changes across multiple tissues in the 10-month-old KI mice^12^ (Fig. S1d). In this analysis, each EHDTS showed the highest degree of overlap with the corresponding brain structure, and was negligible with the transcriptional alterations of non-neuronal tissues. The tissue specificity of the EHDTS was further supported by their overall highest expression levels in the corresponding brain area in the wild-type littermates from the microarray experiments (Fig. 3a). Similarly, this phenomenon was also observed in the human profiles from the GTEx portal, which also revealed a general lower expression of the early signatures in non-brain tissues (Fig. 3b).

To better dissect this selectivity, we determine whether the early alterations in HD showed cellular preference within the brain tissues. To this aim, we examined the presence of cell-enriched markers in the Top-up and Top-down signatures, taking advantage of the TRAP datasets that compile the actively translated transcriptomes in several brain cell subtypes in the cortex, the striatum and the cerebellum^37,38^. We analyzed the top signatures from both animal models per separate, due to the use of different promoters to drive the mHtt transgene expression (ubiquitous in R6/1 and restricted to neurons in N171-82Q mice). Interestingly, the most downregulated genes contained a high number of markers for both excitatory and inhibitory neurons (Fig. 3c–e, Supplementary Table S2). These results were surprisingly similar in both animal models, being the Purkinje cells the only case showing a significant difference (Fig. 3e), which confirmed the documented absence of N171-82Q transgene expression in this cell type^39^ and further validated our selection of differentially expressed genes in early stages of HD. In contrast, very few markers of astrocytes and oligodendrocytes were found in the cortex and the cerebellum (Fig. 3c,e). The differential detection of neuronal and glial markers in the early HD signatures was concordant with the modest effects of mHtt in glial cells compared to neurons^40,41^. EHDTS still retained a high proportion of neuronal markers (Supplementary Table S2), as confirmed with the enriched GO terms and KEGG pathways, mostly related to neuronal functions: synaptic transmission, behaviour, perception, neuroactive ligand-receptor interaction, long-term potentiation and calcium-dependent pathways (Fig. 3f,g).

### The early HD signatures share transcriptionally altered genes with mutants in chromatin-associated proteins

**Figure 4:**
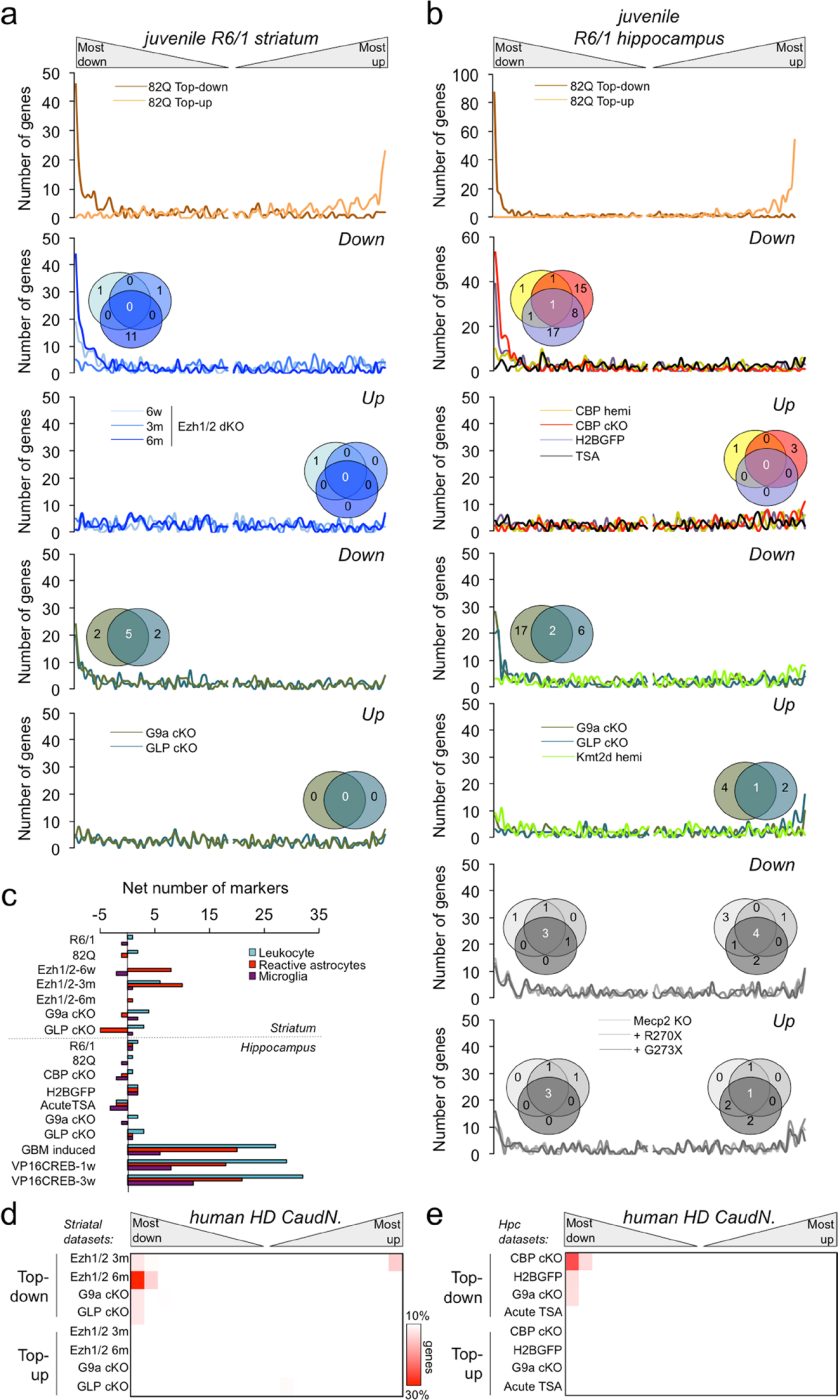
The early HD signatures overlap with the transcriptional profiling for chronic epigenetic dysregulation. **(a**,**b**) Distribution of the top 250 downregulated or upregulated genes as a result of epigenetic/chromatin-related manipulations in mouse striata (**a**) and hippocampi (**b**) across the transcriptome of the juvenile R6/1 striatum and hippocampus respectively, ranked according to the *t* statistic and divided into 100 bins with equal numbers of genes (242). The number of these top DRGs was counted in each bin. Venn diagrams show the number of genes restricted to the EHDTS overlapping with the top deregulated genes in the examined epigenetic-related datasets. cKO, conditional knockout; hemi, heterozygous for the mutation. (**c**), The top250 DRG were investigated for the presence of markers of leukocyte subtypes (T-, B- and NK-cells), activated (reactive) astrocytes and microglia. The net number was calculated as follows: number of markers found in the top upregulation − number of markers in the top downregulation, as a measure of a bona fide immune response. As positive controls, we used datasets from transduced glioblastomas (GBM induced, GSE35917)^89^ and constitutive activation of the transcription factor CREB (VP16-CREB at different time points after transgene expression, GSE21137)^90^. (**d**,**e**) Distribution of the top downregulated or upregulated genes in the striatum (**d**) and the hippocampus (**e**) of the examined epigenetic-related datasets across the caudate nucleus of HD patients^35^, plotted as in Fig. 2.

To gain further insight into the biological implications of the early transcriptional signatures, we extended our meta-analysis using public repositories to transcriptomics studies unrelated to HD. Among the overlapping datasets, we found several gene expression profiles corresponding to mice deficient for chromatin-modifying enzymes, which showed significant fractions of EHDTS among the most downregulated transcripts in the striatum and the hippocampus (*P* < 0.001, Fisher’s exact test; the number of the overlapping genes are shown in the Venn diagrams of Fig. 4). This was the case in the conditional knockouts (cKO) of the histone methyltransferases G9a (*Camk2a-cre; Ehmt2*^*fl/fl*^) and GLP (G9a-like protein, *Camk2a-cre; Ehmt1*^*fl/fl*^)^42^, and the double mutant Ezh1 and Ezh2 (Ezh1^−/−^; Camk2a-cre; Ezh2^fl/fl ^^43^ (significant at older age). The transgenic mice expressing a chimeric protein H2B-GFP (*CaMKII-tTA; tetO-HIST1H2BJ/GFP*), which exhibited severely disrupted chromatin organization^44^, also presented a significant overlap. Keeping in mind the well-established affectation of CBP in HD^19^ and the lack of a defined CBP-dependent transcriptional programme in neurons, we complemented this analysis with a novel microarray experiment in the cKO of this lysine acetyltransferase (*Camk2a-creER*^*T2*^*; Crebbp*^*fl/fl*^). The resulting differential expression in these mice also revealed a high degree of overlap with the EHDTS (*P* < 0.001, Fisher’s exact test). To examine the distribution of the most DRGs in the aforementioned datasets across the whole transcriptome in the striatum and the hippocampus of early HD models, we focused on the R6/1 strain because reproduced better the striatal transcriptional dysregulation (Figs 2b and S1c). In these profiles, the distribution was significantly skewed towards downregulation (*P* < 0.00001 χ^2^ d.f. = 19, compared to random distribution). In contrast, other epigenetic-related datasets did not show a significant overlap (Fig. 4b and Supplementary Fig. S2): the mutants of the methyl CpG reader Mecp2 (*Mecp2*^*−/y*^, *Mecp2*^*−/y*^*; R270X*^*Tg*^ and *Mecp2*^*−/y*^*; G273X*^*Tg*^)^45^, animals acutely treated with the HDAC inhibitor TSA^46^, the homozygous mouse for a gene trap allele of the histone acetyltransferase MYST4/MORF/KAT6B (*Kat6b*^*gt/gt*^)^47^, and the heterozygous knockouts of the histone demethylase Kmt2d/Mll2 (*Kmt2d*^+*/βGeo*^)^48^ and the histone deacetylase HDAC4 (*Hdac4*^+*/−*^^49^). The similarities found with early HD profiles were not due to a shift in the cellular composition of the tissue promoted by inflammatory responses (e.g., gliosis, microglia activation and immune cell infiltration to the detriment of the neuronal subpopulation) (Fig. 4c). Moreover, the overlap between HD signatures and the transcriptional consequences of these epigenetic perturbations was also confirmed in other HD datasets (Supplementary Fig. S3) and, most importantly, in the caudate nucleus profile from patients, mainly for the top DRGs from the Ezh1/Ezh2 KO (*P* < 0.00001 χ^2^ = 201.6, d.f. = 19, Fig. 4d) and the CBP cKO (*P* < 0.00001 χ^2^ = 96.9, d.f. = 19, Fig. 4e). Only in the latter case we observed a concomitant reduction of CBP protein levels in juvenile R6/1 brains (Supplementary Fig. S4). The list of genes common to HD and epigenetic/chromatin dysregulation can be found in Supplementary Tables S3 and S4.

Intriguingly, the gene profiling of the mutants for G9a/GLP and Ezh1/Ezh2 specifically overlapped with transcriptional decreases (Fig. 4a and d for striatum, Fig. 4b and e for hippocampus). Considering that the removal of enzymatic activities associated with repression provokes gene induction^42,43^, this result suggests that early upregulation in HD is not due to a relief of repressive histone methylation. A comparison of the transcriptional dysregulation between the two types of methyltransferase mutants showed that upregulation led to the induction of different sets of genes with different functions (Supplementary Fig. S5). This was not surprising since the mutations affected distinct functional marks: the euchromatic H3K9me2 in the G9a/GLP mutants and the heterochromatic H3K27me3 in the Ezh1/Ezh2 mutants^42,43^. However, downregulation was similar, apparently because of the induction of non-neuronal genes not normally expressed in neurons with repressive roles over the transcriptional programme or with actions that are detrimental to the cell^43^. According to this view, the loss of transcriptional homeostasis in neurons might sensitively affect genes linked to neuronal identity. In fact, downregulation of epigenetic-related genes were highly correlated with downregulation of dopaminergic neuronal markers, in agreement with the presence of a tissular / neuronal-enriched component in the early HD signatures (Fig. 3) and also observed in other HD profiles (Supplementary Fig. S3).

In virtue of these results and considering the importance of epigenetic mechanisms in neuron specification during development, we screened our signatures for markers across developmental stages contained in the Allen Brain Atlas (see Materials and Methods). This analysis confirmed the presence of tissue-enriched markers among the downregulated genes, predominantly corresponding to postnatal stages (Supplementary Fig. S6 and Table S5).

### Selective epigenetic dysregulation is detected in early stages of rapidly progressive Huntington’s disease

**Figure 5:**
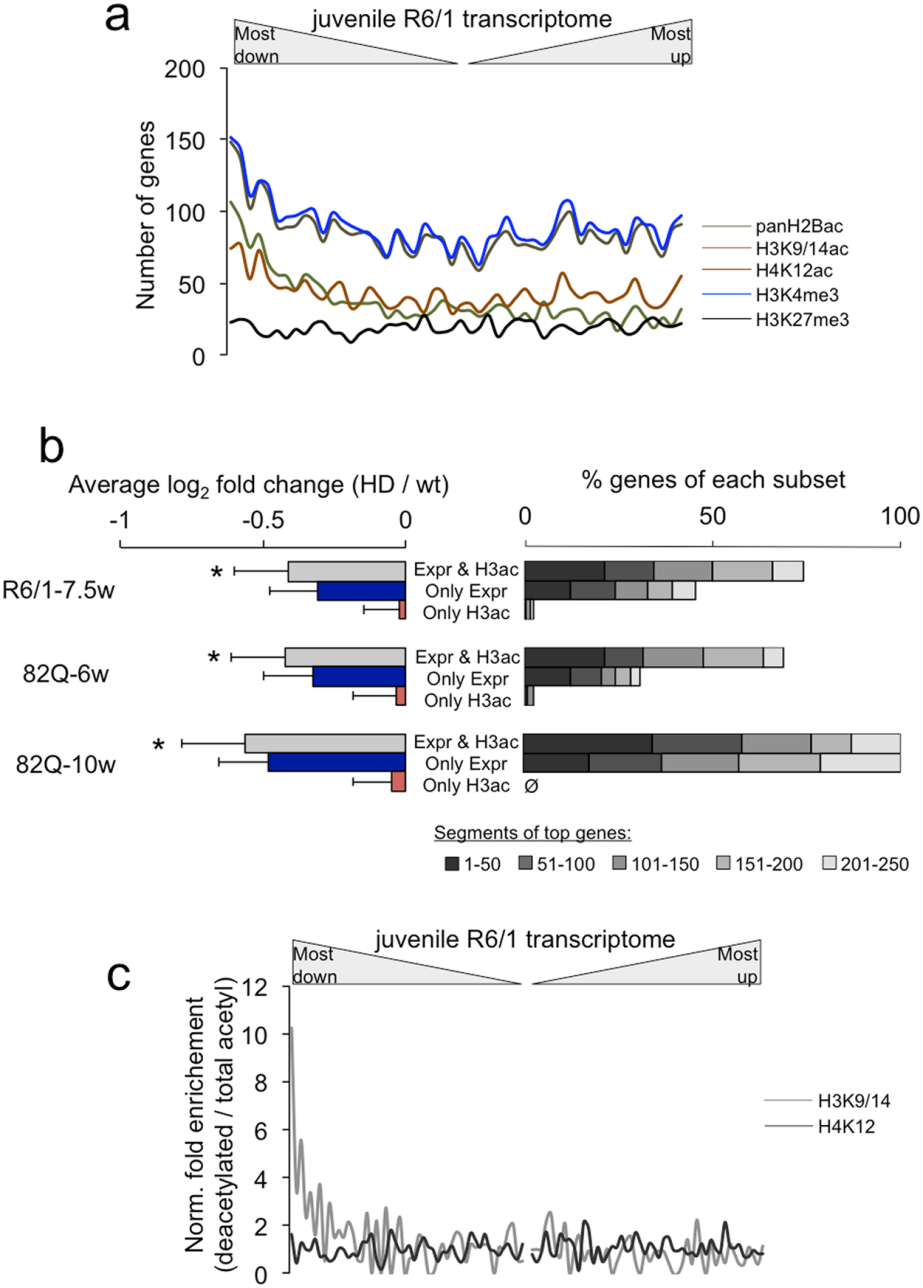
Profiling of histone posttranslational modifications in the early hippocampal HD transcriptome. (**a**) Distribution across the juvenile R6/1 transcriptome of the genes associated with reported histone modifications (panH2Bac, H3K9/14ac, H4K12ac, H3K4me3 and H3K27me in 3–5-month-old mice^46^) at TSS (overlapping with the transcription start sites or comprising the whole gene). (**b**) From a previous study reporting changes in 10-week-old N171-82Q hippocampi^26^, we grouped three types of genes: those showing both gene downregulation and histone H3 deacetylation at the TSS (Expr & H3ac), those showing only downregulation (Only Expr), and those showing only deacetylation (Only H3ac). On the left, fold change in differential expression was averaged within these subsets and represented as the mean ± s.d. for the indicated HD models and ages. *P < 0.05 in all the comparisons between Expr & H3ac and the other groups (Student’s t-test). On the right, the number of overlapping genes in segments of top genes. Compare the top 50 (darkest grey) and top 250 (the whole bar) between the “Expr & H3ac” and “Only Expr” subsets. (**c)** Similar to (**a)** but plotting the fold enrichment (number of genes with deacetylated peaks in 10-week-old N17182Q mice/number of genes with associated peaks from panel (a) per bin).

The resemblance of the early HD profiles with defective chromatin regulation prompted us to investigate the affectation of histone posttranslational modifications in early HD. Taking advantage of the published genomic landscapes of epigenetic marks using similar laboratory conditions as those of the present work^46^, we examined the distribution pattern of the genes associated with histone acetylation (panH2Bac, H3K9/14ac, H4K12ac) and methylation (H3K4me3, H3K27me3) at their transcription start sites (TSS) in the hippocampal R6/1 transcriptome. In contrast to the even distribution of H3K27me3-associated genes, there was a moderate enrichment of genes bearing active expression-associated marks at the top downregulated portion of the R6/1 transcriptome, indicating the main affectation of active genes (Fig. 5a). The most significant profile was retrieved with the genes associated with acetylated histone H2B (*P* < 0.0001, χ^2^ = 101.3, d.f. = 19, compared to H3ac distribution). Because histone H2B is strongly deacetylated in CBP-defective mice and human-derived cells^50–52^, this was consistent with the partial presence of EHDTS in the differential expression profile of the CBP cKO (Fig. 4b) and the incipient change in CBP levels in early R6/1 brains (Supplementary Fig. S4). However, the examined histone covalent modifications tended to converge at the TSS of the hippocampal active genes^46^ to constitute *cis*-regulatory modules^53^, therefore they provided highly redundant information.

**Figure 6:**
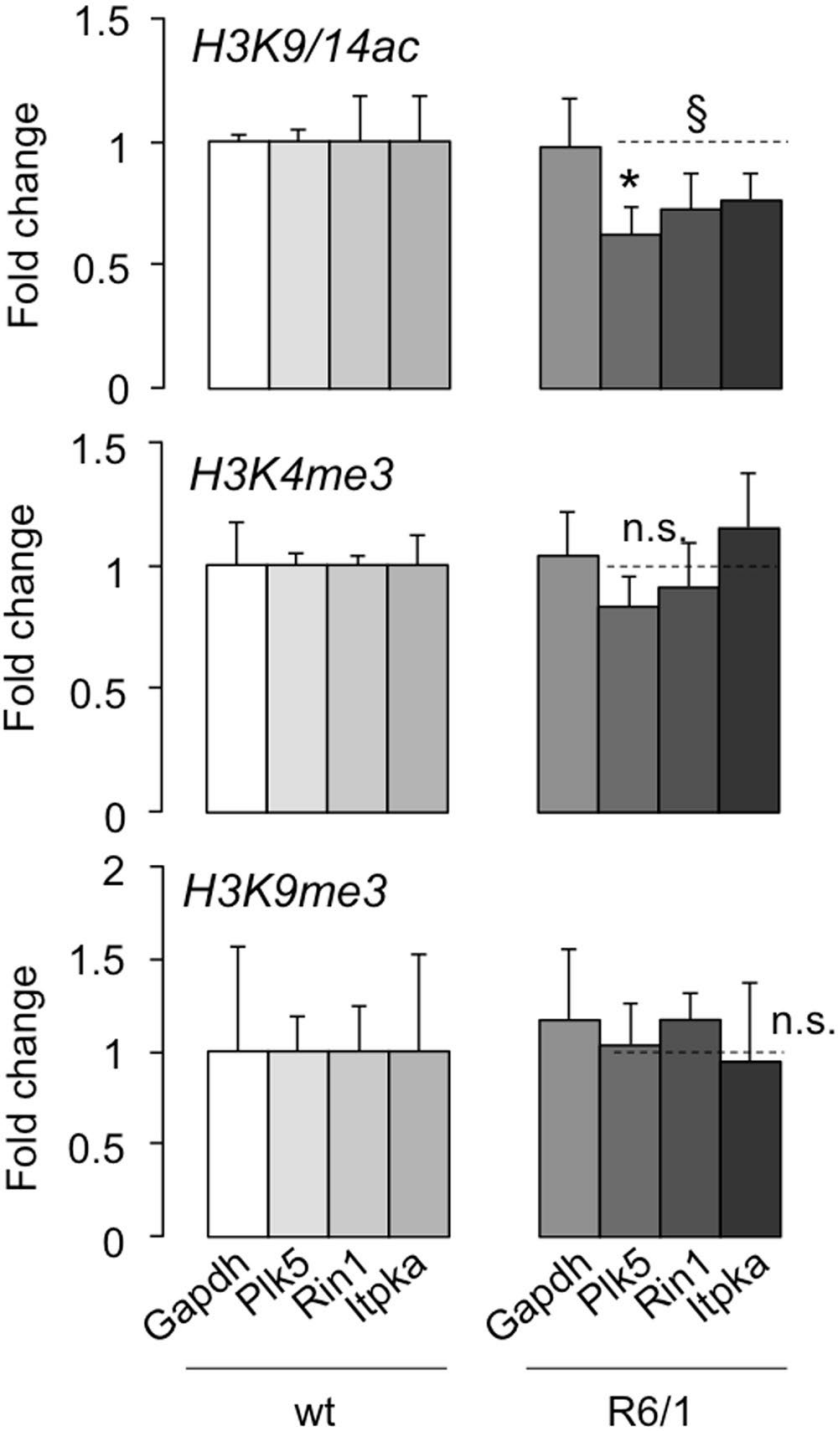
Histone acetylation is selectively impaired in the early R6/1 cortex. ChIP assay in the prefrontal cortex of R6/1 and wild-types littermates of the indicated histone modifications followed by qPCR of selected TSS regions: *Gapdh* (non-changing active gene) and the genes of interest *Plk5*, *Rin1* and *Itpka*. n = 4 for each genotype. Data are expressed as the mean ± s.e.m. *P < 0.05 for *Plk5*, ^§^*P*<0.05 considering the three genes altogether (Student’s t-test). n.s., not significant.

Further evaluation of the transcriptionally altered genes in the hippocampus of 10-week-old N171-82Q mice^26^ revealed that they showed some degree of affectation in 6-week-old animals, being in general more severe in those genes with concomitant histone H3 deacetylation of lysines 9 and 14 (compare the fold change and rank between “Expr & H3ac” and “Only Expr” in Fig. 5b). This behaviour was reproduced in the R6/1 hippocampus (Fig. 5b). In these mice, one third of the reported genes with histone H3 deacetylation were concentrated towards downregulation, in contrast to the distribution pattern of genes with H4K12 deacetylation (Fig. 5c). Of these, 25 genes were present in the hippocampal EHDTS (Supplementary Table S4). This phenomenon was not exclusive of the hippocampus as the striatal EHDTS also contained a high proportion of genes reported to be associated with decreased K27 acetylation and/or H3K4 methylation of histone H3^27,29^ (Supplementary Fig. S7). Based on these results, we asked whether this was indicative of a general altered chromatin architecture that might contribute to the pathological transcriptional profile in HD. To answer this question, we used a battery of antibodies against histone H3 modifications associated with states of gene activation (H3K4me3 and H3K9/14ac) and inactivation (H3K9me2/3 and H3K27me3) in upper layers of the cortex, the striatum and the CA1 subfield of the hippocampus in the R6/1 strain in a fully symptomatic age (20-week-old) to ensure the observation of potential changes. Supplementary Fig. S8 depicts the representative case of the striatum. Apart from an overall reduction in the nuclear size of R6/1 cells, the staining pattern of euchromatin and heterochromatin markers and the distribution and number of nuclear bodies were indistinguishable between mutant and wild-type littermates. Moreover, we observed the absence of differences in the global levels of histone H3 acetylation and K4 trimethylation, in agreement with our previous findings^36^. In conclusion, these results suggested that the nuclear architecture in mHtt-expressing neurons was not dramatically altered in terms of chromatin condensation and histone localization, even in advanced stages of disease. We also examined the local occupancy of modified histones by chromatin immunoprecipitation assays in cortical samples. In contrast to a generalized perturbation, we only experimentally confirmed the significant deacetylation of histone H3 in early stages of the pathology for the TSS of *Plk5*, although there was a general trend in defective histone acetylation in other TSSs (*Rin1* and *Itpka*) (Fig. 6). Neither demethylation of lysine 4 nor hypermethylation of lysine 9 were detected in the examined loci (Fig. 6). Therefore, there was a highly selective disruption of histone covalent modifications associated with transcriptional dysregulation in early HD.

## Discussion

In this work, we dissected the transcriptional profiles of relevant brain areas in mHtt-expressing mice at a juvenile age. These early signatures, linked to the gain-of-function component of HD, were also observed in the top altered genes in other HD models, including KI mice that also model the loss-of-function component^12,32,33,54^, and in post-mortem tissues of HD patients^34,35^. Thus, we confirmed the consistency of the altered gene expression pattern in HD (first found by Luthi-Carter and colleagues^55^ and validated by others in subsequent studies) and enhanced the information regarding less explored ages (i.e., puberty) and brain areas (e.g., hippocampus). This consistency was more obvious in gene downregulation (as previously described^26,56^), whereas upregulated genes served as negative controls in most of the analyses performed in the present work.

Noticeably, the early signatures contained an important tissue-enriched component (Figs 3a,b, S1d and S6), reflecting the transcriptional distinction between neuronal subtypes across brain areas (Fig. 3c–e). This conclusion is reminiscent of a previous report that dissected the tissue-specific and commonly affected pathways between the striata and cerebella of pre-symptomatic KI mice, independent of the robustness of altered expression^57^. To understand this tissue selectivity, an unbiased meta-analysis revealed striking similarities between the transcriptional dysregulation as a result of long-term chromatin perturbations and the HD signatures in mouse models and patients (Fig. 4), suggesting that the tissular / neuronal specificity were regulated by chromatin remodelling, in accordance with the general role of epigenetics in specification and maintenance of neuron identities. This is well exemplified by the deletion of G9a in striatopallidal Drd2^+^ neurons, which causes the switch to the striatonigral Drd1^+^ transcriptional program^58^. Moreover, a previous study identified a decrease in the deposition of H3K27ac, a feature of super-enhancers or large enhancers regions that drive cell-type-specific expression, which were associated with the downregulation of striatal-enriched genes in the R6/1 strain^27^. Because this resemblance was specific for downregulation, independent of the association of the mutated enzymatic activities with gene activation or repression, we should not consider *a priori* that these activities were actually deficient in HD; in fact, we were unable to detect a reduction in the levels of the methyltransferases Ezh1, Ezh2, G9a and Hdac1 (Supplementary Fig. S4) to initially support their involvement in transcriptional dysregulation in prodromal stages. By analogy to these mutants, a defective epigenetic environment in HD seems to affect the neuronal transcriptional programme by indirect mechanisms, with the tissue-enriched genes among the most susceptible^20^. For instance, Ezh1/Ezh2 and G9a/GLP enzymes played substantially different roles as suggested by the upregulation of largely distinct subsets of genes while sharing the downregulation of canonical striatal genes (Supplementary Fig. S5), as confirmed by the analysis of anatomically enriched genes (Supplementary Fig. S6), despite derepressing an important proportion of non-neuronal genes in both cases. This principle can also be applied to the hippocampus, as the accumulation of chimeric protein H2B-GFP in neuronal heterochromatin disrupted the general nuclear organization but selectively affected representative genes of the hippocampus (Supplementary Fig. S6a) related to cognition^44^.

Previous reports proposed that the altered activities of certain transcription factors deregulated the expression of downstream enzymatic modulators and caused aberrant epigenetics (as in the case of H3K9 methyltransferases^19^), but the view proposed here predicts that an aberrant chromatin environment affects the expression of transcription factors relevant for neuronal identity and functioning. As plausible candidates, the early signatures identified the upregulation of cortical Prdm8, which can form a repressor complex in neurons^59^, and SoxC members (Sox4 in the cerebellum, and Sox11 in the cortex and the striatum) that are involved in CNS developmental processes, including neuronal subtype specification^60^. Transcriptional dysregulation of mature postnatal genes by well-known developmental factors may open a new avenue to explore the possible effects of mHtt during prenatal and perinatal development^61^. Of note, some few genes tightly associated with HD (e.g., Pde10a in striatum) were highly expressed in embryonic stages (Supplementary Table S5).

The epigenetic impairment in early HD is still undisclosed. A candidate that may exert a direct effect over chromatin dysregulation is CBP, in concordance with the well-established disruption of its activity by sequestration and inhibitory interaction with mHtt, the restoration of which leads to the rescue of phenotypical and molecular traits related with polyQ pathology^19^. Moreover, the juvenile R6/1 brain exhibited a modest reduction of CBP levels (Supplementary Fig. S4), and striatal downregulation in symptomatic R6/1 has been associated with deacetylation of the aforementioned H3K27^27^, an important CBP substrate. The interplay between CBP and other chromatin regulatory protein may enhance our understanding of HD transcriptional dysregulation. For example, H3K27ac-marked super-enhancers can interact with broad H3K4me3 domains^62^, which were also linked with downregulation of neuronal genes in HD^29^. In agreement with histone acetylation impairment, we were able to extend the locus-specific deacetylation of lysines 9 and 14 of histone H3 in symptomatic mice^26,36^ to an earlier stage of the pathology (Fig. 6). In contrast, the association with HD signature was not observed with the profile of the mutant mouse for another lysine acetyltransferase, Kat6b (Supplementary Fig. S2). Despite showing histone H3 hypoacetylation and altered transcription in these mice^47^, this negative overlap with HD signatures promotes the prominence of CBP in the HD transcriptional dysregulation. Further studies are still necessary to precisely identify the chromatin-modifying enzymatic activities that become defective before overt manifestations of the phenopathology in order to delineate the first molecular alterations in HD.

## Methods

### Animals

The transgenic strains B6.Cg-Tg(HDexon1)61Gpb/J (aka R6/1)^63^ and B6C3-Tg(HD82Gln)81Dbo/J (aka N171-82Q)^39^ were acquired from Jackson Laboratories. CBP^fl/fl^ and CaMKII-creER^T2^ mice have been previously described^64,65^. Tamoxifen was administered to 2-month-old CBP^fl/fl^ mice, either carriers or non-carriers of the cre recombinase transgene^50^. N171-82Q mice were maintained in a DBA and C57BL/6 J mixed background (50:50) because they cannot be bred in a pure C57BL/6J background. All other mice were maintained in a pure C57BL/6 J background. Mice were kept on a 12-h light/dark cycle with food and water provided *ad libitum*. Experimental protocols were approved by Institutional Review Boards (Comité de Bioseguridad y Bioética del Instituto de Neurociencias, Comité de Ética de Experimentación Animal - Órgano Habilitado de la Universidad de Cádiz) and authorized by Subcomité de Bioética CSIC and Dirección General de la Producción Agrícola y Ganadera de la Junta de Andalucía according to European and regional regulations.

### RNA extraction, RT-qPCR and microarrays

Total RNA was extracted using the TRI reagent (Sigma-Aldrich) and reverse transcribed to cDNA using the RevertAid First-Strand cDNA Synthesis kit (Fermentas). qPCR was performed in the Qiagen Rotor-Gene Q Detection System using PyroTaq EvaGreen qPCR Mix Plus (Cmb-Bioline). For all the kits, the manufacturers’ recommendations were followed. Each independent sample was run in duplicate and normalized using *Gapdh* levels. The sequences of all primer pairs are shown in Supplementary Table S6.

For the microarray experiments, RNA from 3 to 4 males of the same genotype were pooled and cleaned up using the RNeasy Mini Kit (Qiagen). RNA samples were obtained at different ages: 6 weeks old (N171-82Q), 7.5 weeks old (R6/1) and 4 to 4.5 months old (CaMKII-creER^T2^: CBP^fl/fl^). Time sampling was different in HD mice to compensate for the differential progression of the pathological phenotype, as evidenced by the normal age of premature death ( >24 and >30 weeks old in N171-82Q and R6/1, respectively). Three independent pooled samples per genotype and brain area were hybridized to Mouse Gene 1.0 ST expression arrays (Affymetrix). Microarray data were background corrected, normalized, summarized, and statistically analysed using R and Bioconductor^66^. Briefly, Mouse Gene 1.0 ST arrays were read using the *oligo* package, with the RMA algorithm as the normalization method, and moderated t tests were calculated using the *limma* package^67^. To facilitate subsequent analyses we removed non-annotated and redundant probesets and we ranked the resulting profiles according to the significance of change (*t* statistic) for each pair-wise comparison. The Top-down and Top-up subsets were selected from the 250 genes showing the most extreme values of the *t* statistic. In this manner, we avoided any potential bias from varying degrees of dysregulation (i.e., different length of the resulting lists of differentially expressed genes). These files can be downloaded from the Gene Expression Omnibus (GEO) database using the accession number GSE107613.

### Meta-analysis with external genome-wide datasets

Most of the datasets used in the present study were obtained from the public repositories ArrayExpress (https://www.ebi.ac.uk/arrayexpress/) and GEO (https://www.ncbi.nlm.nih.gov/gds/) using the following search words: “cortex”, “cortical”, “striatum”, “striatal”, “hippocampus”, “hippocampal”, “cerebellum” and “cerebellar”, combined with the corresponding IDs for the Affymetrix formats Mouse Gene 1.0 ST and Mouse Genome 430 2.0. We focused on these formats to balance the number of datasets (as there were relatively few experiments in the ST format) and the number of common genes between platforms (due to the variety of formats with different gene representation and nomenclature). The downloaded CEL files were processed in a similar manner as the early HD arrays, except for the use of the *affy* package^68^ to read files derived from the Affymetrix 430 2.0 format. For the meta-analysis, we considered: i) annotated probe sets, ii) the most significant probe set in genes with multiple probe sets, and iii) common genes between both formats, leaving a final number of 17328 genes. Differential expression was calculated using the control condition (e.g., wild-type and/or vehicle) in each time of sampling for all the possible pair-wise comparisons within the same accession number. The top 250 with the lowest (downregulated) and highest (upregulated) *t* statistic values were compared with the EHDTS in each brain area. The most relevant accession numbers used in the preparation of figures were: GSE7958, GSE9803, GSE9804, GSE10202^55^, GSE9038^57^, GSE18551^69^, GSE29681^70^, GSE32417^71^, GSE50379^72^, GSE62210^73^, GSE70656^74^ for HD (Fig. S1a,b); GSE7707, GSE19534^75^, GSE20547^76^, GSE24829^77^, GSE52584^78^, GSE60413^79^ for PD (Fig. S1b); E-MEXP-1313, E-MEXP-1314^80^, GSE9914^81^, GSE55177^82^, GSE61908^83^, GSE72176 for SCA (Fig. S1b); GSE19402 for *Camk2a-cre; Ehmt2*^*fl/fl*^ and *Camk2a-cre; Ehmt1*^*fl/fl* ^^42^; GSE22969 for *Kat6b*^*gt/gt* ^^47^; GSE30880 for *Crebbp*^+*/−* ^^50^; GSE38218, GSE38219 for *Hdac4*^+*/−* ^^49^; GSE42987 for *Mecp2*^*−/y*^, *Mecp2*^*−/y*^*; R270X*^*Tg*^ and *Mecp2*^*−/y*^*; G273X*^*Tg* ^^45^; GSE44868 for acute TSA treatment^46^; GSE48437 for *CaMKII-tTA; tetO-HIST1H2BJ/GFP*)^44^; GSE84243 for Ezh1^−/−^; Camk2a-cre; Ezh2^fl/fl ^^43^; and GSE90836 for *Kmt2d*^+*/βGeo* ^^48^ (Figs 4 and S2). As in previous studies^84–86^, the distribution of the top DRG was plotted across the whole transcriptome of reference, once ranked by the *t* statistics retrieved in the differential expression analysis, and divided into bins of equal number of genes.

To obtain specific markers from neurons and glia (Fig. 3c–e), we used the datasets obtained after affinity purification of polysomal mRNAs from transgenic mice expressing EGFP-tagged ribosomal protein L10a in a variety of genetically defined cell populations (GSE13379)^37,38^. Given a cellular subtype in the cortex, the striatum or the cerebellum, CEL files were reanalysed using *affy* and *limma*, and the genes selected were the most differentially expressed in all the possible pair-wise combinations with other cellular subtypes within the same brain area. The top 100 genes in all the comparisons were considered as specific markers. Datasets from the neurons of cortical layers 5a, 5b and 6 were pooled to retrieve the desired number of markers with a significant adjusted *P*-value < 0.05, as we did with Drd1 and Drd2-expressing striatal cells. Using the same approach, we also obtained the specific markers for reactive astrocytes (commonly changing genes after 1d of activation with either LPS or middle cerebral artery occlusion) compared to unstimulated astrocytes (GSE35338)^87^, and microglia and a mix of B, T and NK-cells (“leukocyte”) compared to the striatum/hippocampus profiles (GSE10246)^88^. As positive controls of inflammation and immune response, we used the datasets from induced glioblastomas (GBM induced, GSE35917)^89^ and constitutive activation of the transcription factor CREB (VP16-CREB at different time points after transgene expression, GSE21137)^90^ (for Fig. 4c).

In addition, we also employed whole genome-wide data as reported in the original publications: post-mortem brain transcriptomes from HD patients (caudate nucleus and cerebellum, Table S1 from^35^; prefrontal cortex BA9, S1 File from^34^) (for Figs 2, 4d–e), RNA-seq of HD knock-in mice with different numbers of CAG repeats (Supplementary Table 1 from^12^) (for Figs S1c–d, S3b), RNA-seq and ChIP-seq from 30-week-old R6/1 striatum (Supplementary Table 1 and 3 from^27^) (for Figs S3a and S7), genomic detection of histone covalent modifications in the wild-type mouse hippocampus (Supplementary Data Set S1 from^46^), differential acetylation of histones H3K9/14 and H4K12 in the hippocampi of 10-week-old N171-82Q mice (Supplementary material from http://in.umh.es/barcolab/datasets.html26) (for Fig. 5) and downregulated and hypomethylated genes in the R6/2 striatum (Dataset S2 from^29^). We also retrieved the top 43 murine genes (imposed by the number of genes for E15.5 in the central subpallium) from the “Anatomic Search” tool at the website http://developingmouse.brain-map.org within the Allen Brain Atlas (http://www.brain-map.org) (for Fig. S6a). Enriched genes for each developmental stage were defined based on the *in situ* hybridization signals for the central subpallium or the medial pallium in contrast to the signals from the telencephalic vesicle; the results of the comparison were expressed as the ratio of the sum of expressed pixel intensities (see the documentation of the website for further details). Finally, we used the following portals to extract human transcriptomes: GTEx (www.gtexportal.org)^91^ and BrainSpan (www.brainspan.org) that contained the profiles across human tissues and human lifespan, respectively. Either the Transcripts Per Million (TPM) or Reads Per Kilobase Million (RPKM) were collected for the genes of interest, converted to log2 and averaged for the subset of genes (for Figs 3b and S6b).

### Additional bioinformatics

Overlapping genes between multiple lists of genes were retrieved using Venny (http://bioinfogp.cnb.csic.es/tools/venny/) and Venn Diagrams (http://bioinformatics.psb.ugent.be/webtools/Venn/). The HCOP tool of the HUGO Gene Nomenclature Committee (HGNC) was used to search gene orthologues (http://www.genenames.org/cgi-bin/hcop) to improve the comparisons between human and mouse expression data. Enrichment for Gene Ontology (GO) terms related with biological processes was calculated using Webgestalt 2013 (http://www.webgestalt.org/webgestalt_2013/)^92^, using the mouse genome as a reference.

### Immunodetection and ChIP analyses

Protocols for immunohistochemical and Western blot analyses in brain tissues are described elsewhere^93,94^. The antibody against the pan-acetylated histone tails of H3 (K9/14) was produced in-house^93^. Other antibodies were H3K9me2 (ab1220), H3K9me3 (ab8898), HP1-α (ab77256), H3K27me3 (ab6002), HDAC1 (ab109411), H3 (ab176842, Abcam), H3K4me3 (07-473), H2B (07–371), CBP (sc-7300), ENX-1 (Ezh2, sc-137255), ENX-2 (Ezh1, sc-515817), G9a (sc-515726, Santa Cruz Biotechnologies), biotinylated and HRP-conjugated (Sigma-Aldrich Química S.A.) and Alexa Fluor 488 and 594 (Molecular Probes, Invitrogen) as secondary antibodies. All quantifications were performed using ImageJ software.

For the ChIP assays, mice were sacrificed by cervical dislocation, and the prefrontal cortices were dissected. The ChIP-qPCR assays were then performed as previously described^50^ with minor modifications. Briefly, four independent tissue samples per condition were obtained by pooling the cortices of three mice. After fixation with 1% formaldehyde for 10 min, glycine quenching for 5 min and nuclei isolation, chromatin was sonicated with a Sonifier SFX150 (Branson) and immunoprecipitated with 1:200-1:250 dilutions of the antibodies against H3K9/14ac (07-599), H3K4me3 (07-473, Millipore) and H3K9me3 (ab8898, Abcam). Real time PCR of the immunoprecipitated chromatin was performed as described for RT-qPCR using specific primer pairs that were close to the TSS (see Supplementary Table S6 for the sequences) using the SYBR Premix Ex Taq (TIi RnaseH Plus) (Takara, Clontech) following the manufacturer’s recommendations. The percentage of immunoprecipitated DNA over input DNA was determined. Once we had ensured the performance of the procedure with the resulting values from the TSS of *Gapdh* (housekeeping gene) and *Hbb-bh1* (foetal haemoglobin, inactive in adulthood), we calculated the fold change between genotypes.

## Electronic supplementary material

Supplementary Figures S1-S8

Supplementary Tables S1-S6
