# Supplementary material for: Early alteration of epigenetic-related transcription in Huntington’s disease mouse models
Source: Sci Rep. 2018 Jul 2;8:9925. doi: 10.1038/s41598-018-28185-4 (PMC6028428; doi:10.1038/s41598-018-28185-4)

Supplementary Figures to "Early alteration of epigenetic-related transcription in Huntington's disease mouse models" Irati Hervás-Corpión, Deisy Guiretti, Manuel Alcaraz-Iborra, Román Olivares, Antonio Campos-Caro, Ángel Barco, Luis M. Valor

Supplementary Legends

Supplementary Figure S1. The early striatal signature is present in the transcriptomes of other HD models. a, Table of the most frequent genes in striatal HD datasets (at least appearing in 40% of them). "In EHDTS" and "absent" refers to their presence or absence in the the striatal EHDTS, respectively. **b,** Percentage of the EHDTS from the striatum (upper) and the cerebellum (lower) represented in publicly available datasets related to HD, Parkinson's disease (PD) and spinocerebellar ataxias (SCA). The ataxia most resembling HD was SCA17 (E-MEXP-1313 and GSE72176), caused by an expansion of CAG/CAA repeats in the TATA-binding protein (*Tbp*) gene. Data are represented as the mean ± s.d. Conc and Disc, transcriptional changes that are concordant (in the same direction of change) or discordant (in the opposite direction), respectively. **c,** Percentage of striatal EHDTS across the whole striatal transcriptome of KI of different ages and CAG repeats, ranked according to *t* statistics and divided for simplification into 10 bins of equal numbers of genes (1710). We counted the number of dowregulated EHDTS ("EHDTS DOWN") and the Top-down and Top-up genes from each mouse model ("R6/1", "N17182Q") in each bin. **d,** Percentage of EHDTS from each brain area overlapping with the most downregulated genes (first bin of **c**) across brain and non-brain tissues from 10-month-old KI-Q175 mice. Hypothal, hypothalamus; Thalam, thalamus; C. callosum; corpus callosum; adip., adipose.

Supplementary Figure S2. The available epigenetic-related profiles in the cortex do not overlap with the early HD signature. Distribution of the top 250 downregulated or upregulated genes as a result of epigenetic/chromatin-related manipulations in mouse cortices across the juvenile cortical R6/1 transcriptome (ranked according to the *t* statistic and divided into 100 bins of equal numbers of genes (242)). The number of these top DRGs was counted in each bin. Venn diagrams show the number of genes restricted to the EHDTS overlap. Kat6b hypomorphic allele of Kat6b; HDAC4 hemi, heterozygous knockout for HDAC4. The action of HDAC4 was proposed to be histone-independent (Mielcarek *et al. PLoS biology*, 11, e1001717, 2013).

Supplementary Figure S3. External HD signatures also overlap with the transcriptional profiling for chronic epigenetic dysregulation. a, Distribution of the top 250 downregulated or upregulated genes as a result of epigenetic/chromatin-related manipulations in mouse striata across the transcriptome of 30-week-old R6/1 striatum (Achour *et al. Human molecular genetics* 24, 3481-3496, 2015), ranked according to the *t* statistic and divided into 100 bins with equal numbers of genes (263). The number of these top DRGs was counted in each bin. dKO, double knockout; cKO, conditional knockout. **b,** Percentage of striatal EHDTS across the whole striatal transcriptome of KI of different ages and CAG repeats (Langfelder *et al. Nature neuroscience* 19, 623-633, 2016), ranked according to *t* statistics and divided for simplification into 10 bins of equal numbers of genes (1710). We counted the number of the top downregulated genes as a result of epigenetic/chromatin-related manipulations in mouse striata in each bin. Cholinergic (ACh) and dopaminergic (DA) markers were the top100 genes retrieved in the TRAP analysis and used in Figure 3c-e.

Supplementary Figure S4. CBP protein levels are slightly reduced in prodromic R6/1 brains. Western blot analysis of Ezh1, Ezh2, G9a, Hdac1, histone H3 and CBP in the brains of R6/1 mice and wild-type littermates. **a, c,** Representative blots. **b, d,** Quantification. n = 5 for wt, n = 5 for R6/1. Data are expressed as the mean ± s.e.m. *, $P < 0.05$ comparing genotypes within the same brain area; §, $P < 0.05$ comparing genotypes in all the brain areas (Student's t-test).

Supplementary Figure S5. Ablation of different histone methyltransferases leads to downregulation of striatal genes. a, Distribution across the 6-month-old double mutant Ezh1/Ezh2 transcriptome of the top 250 downregulated or upregulated genes in the double mutant at the age of 6 weeks (6w) and 3 months (3m), the conditional knockouts G9a and GLP and the HD models (R6/1 and 82Q). We plotted the distribution as in Fig. 4 and S2. As expected, there was an increasing gene induction across the different ages of the double mutant. The number of genes in the most downregulated part of the transcriptome was highly similar in most of the conditions. **b-c,** GO analysis of the top250 downregulated (**b**) and upregulated (**c**) genes in the histone methyltransferases knockouts; terms with an adj. *P*-value <0.05 (Webgestalt) were manually grouped. Downregulation showed more similarities at the functional level than upregulation.

Supplementary Figure S6. Anatomical markers of the developing mouse brain in HD and epigenetic-related profiles. a, Overlap between the top enriched markers in each developmental stage and brain structure in mouse (according to Allen Brain Atlas (http://developingmouse.brain-map.org)) and the top genes in HD and chromatin-related datasets. **b,** We confirmed the prominence of HD signatures in postnatal ages by examination of the expression levels of striatal (upper) and hippocampal (lower) EHDTS in

human striatum and hippocampus across different ages (according to BrainSpan, Allen Brain Atlas). The comparison with the expression profile of classical markers for mature neurons (Calb1/Calbinding D28, Calb2/Calretinin, Dlg4/PSD-95, Eno2/NSE, Map2, Nefh, Nefl, Nefm, Rbfox3/NeuN, Syp) and for precursors and immature neurons (Ascl2/MASH2, Cdh1/E-cadherin, Cdh2/N-cadherin, Dcx, Eomes/Tbr2, Hes1, Hes3, Hes5, Nes, Neurod1, Notch1, Ocln, Pax6, Tbr1, Slc1a3/EAAT1, Sox2, Stmn1, Tnc, Tubb3, Vim) revealed that the EHDTS followed the same pattern is the mature neuronal markers. Pearson coefficients indicate the correlation with EHDTS expression profile across human brain development.

Supplementary Figure S7. The striatal early signatures contain genes with defective histone modifications. Overlap between the early signatures (striatal EHDTS and Top-down genes in N171-82Q and R6/1) and downregulated genes with altered histone modifications as described in other studies (H3K4me3, Vashishtha *et al. PNAS* 110, E3027-36, 2013; H3K27ac, Achour *et al. Human molecular genetics* 24, 3481-96, 2015). In red, genes altered in the methyltransferases knockouts (Ezh1/2 6m, G9a cKO, GLP cKO; Supplementary Table S4); in italic, genes consistently altered in HD models (Supplementary Fig. S1a). The EHDTS contained the higher proportion of genes previously reported to be associated with hypoacetylation and/or hypomethylation compared to the Top-down genes for each model.

Supplementary Figure S8. Global chromatin organization is not altered in the striatum of R6/1 mice. a, Representative immunostaining images of striatal R6/1 and wild-type littermates against marks linked to repressed genes (H3K9me2, H3K9me3, H3K27me3 and heterochromatin protein HP1-α and active genes (H3K4me3 and H3K9/14ac). **b,** The immunostaining intensity was quantified in those marks showing a

diffuse subcellular pattern that allowed measurements of the whole nucleus. **c,** Number of nuclei (to infer number of cells) and nuclear bodies (to infer the nuclear architecture) per frame were counted according to DAPI staining. **d,** Area and perimeter of striatal nuclei. N = 4 (mice) and n = 135 (cells) for wt, N = 6 (mice) and n = 257 (cells) for R6/1. Data are expressed as the mean ± s.e.m. *, $P < 0.05$ between genotypes (Student's t-test).

Supplementary Figure S1. The early striatal signature is present in the transcriptomes of other HD models

a

Down (in EHDTS)	Down (absent)	Up (in EHDTS)	Up (absent)
Arpp19	Nrep	Lrrn3	Abca8a
Cnr1	Ryr1	Pcdhb9	Acy3
Coch	Slc4a11	Pol2ra	Arid5b
Ddit4l		Rgs19	Fat1
Gpr83		Sox11	Pcdh20
Gsg1l			Pcdhb21
Lynx1			Pde4d
Neto2			Psme1
Pde10a			Sfmbt2
Penk			Smoc11
Phex			
Prima1			
Rgs4			
Rspo1			
Scn4b			
Strn			
Tbc1d8			

b

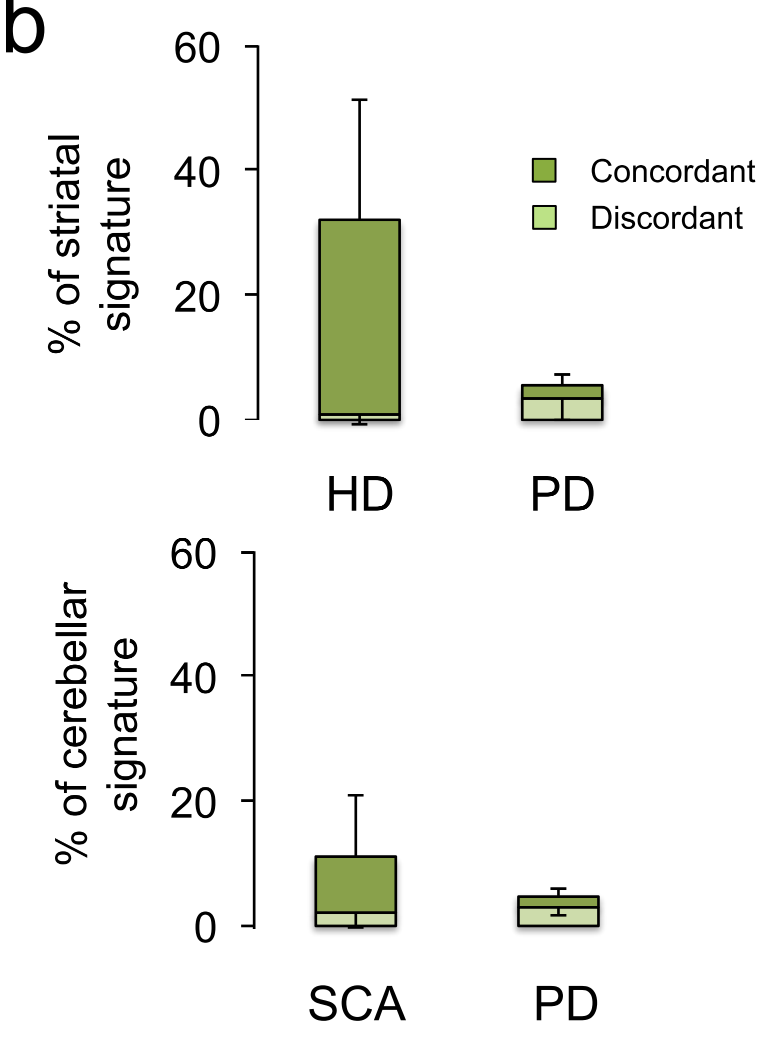

c

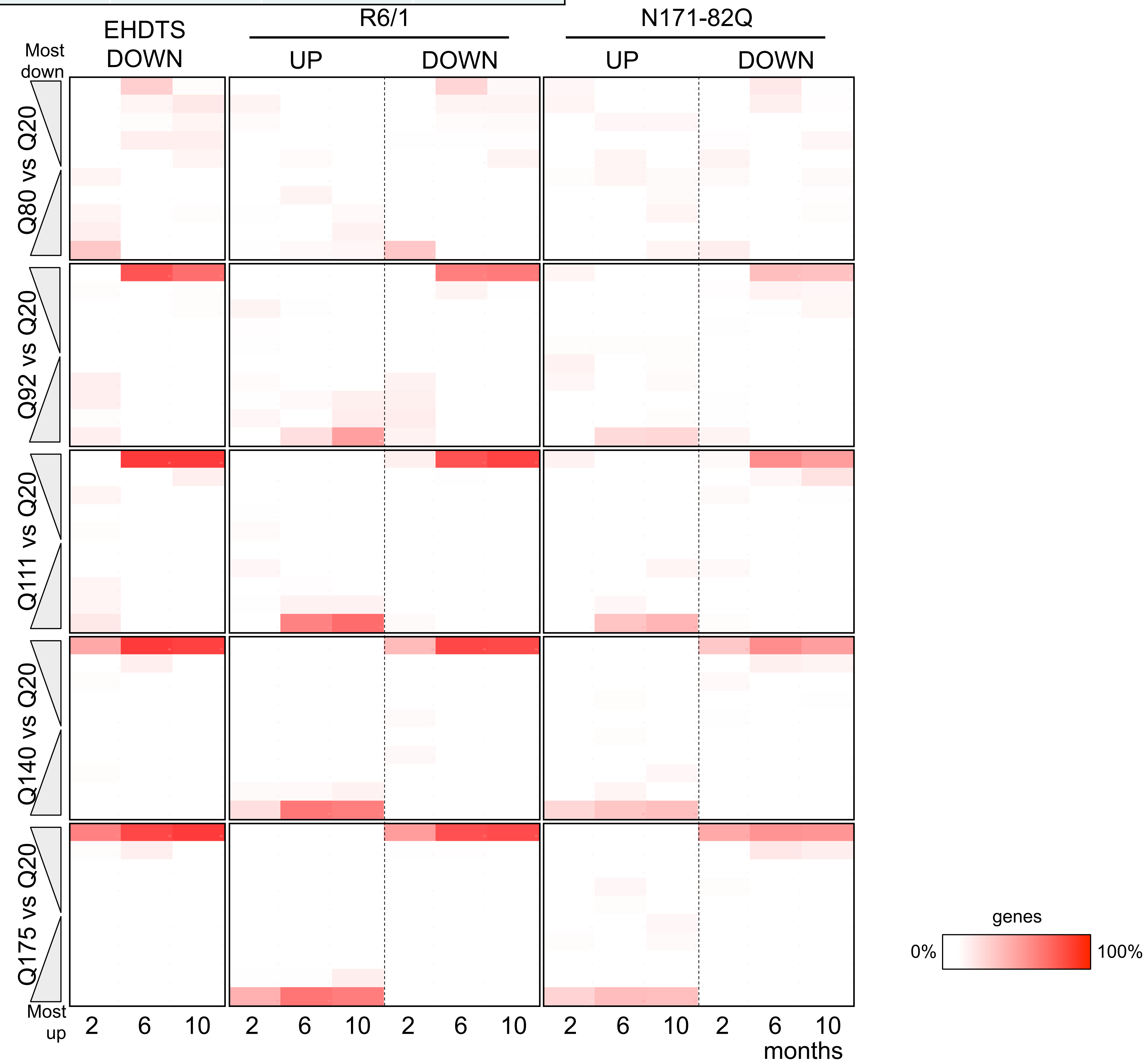

d

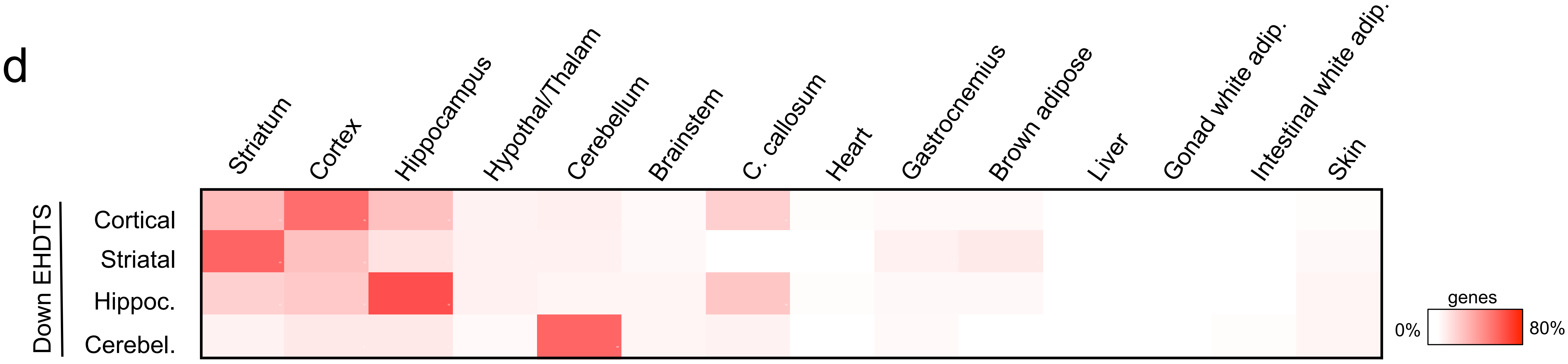

Supplementary Figure S2. The available epigenetic-related profiles in cortex do not overlap with the early HD signature

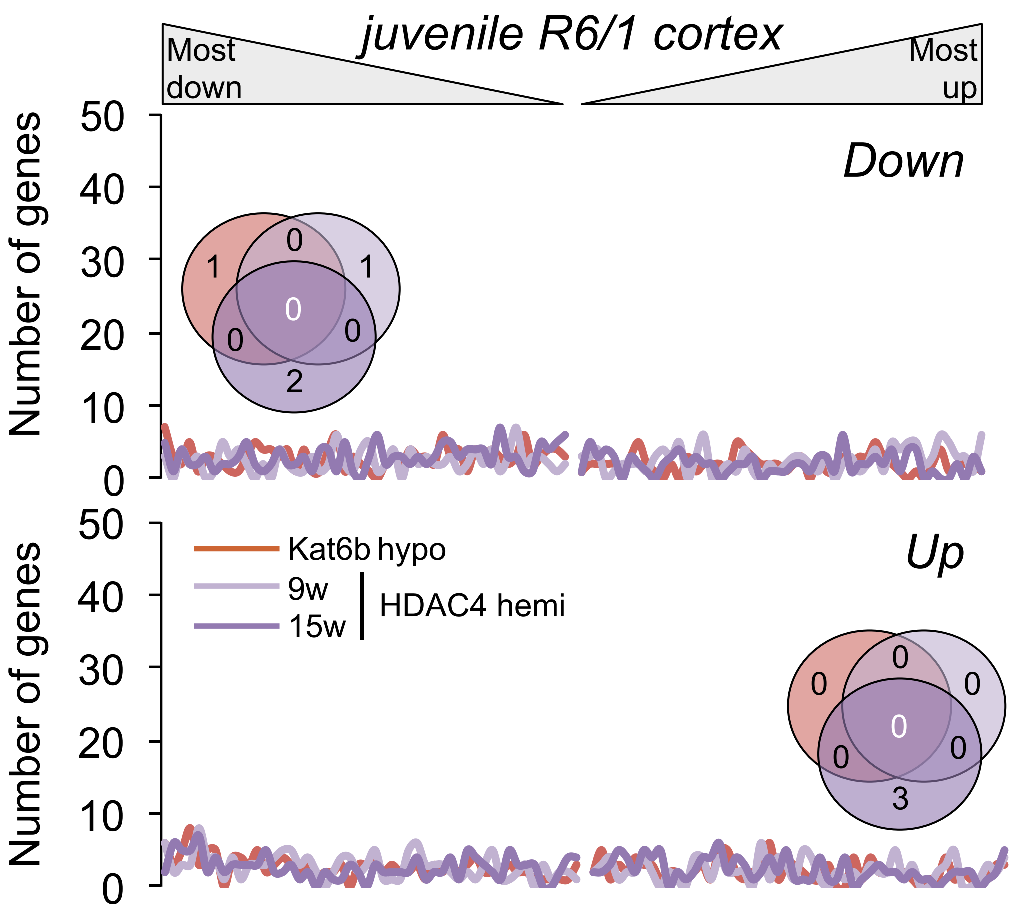

Supplementary Figure S3. External HD signatures also overlap with the transcriptional profiling for chronic epigenetic dysregulation

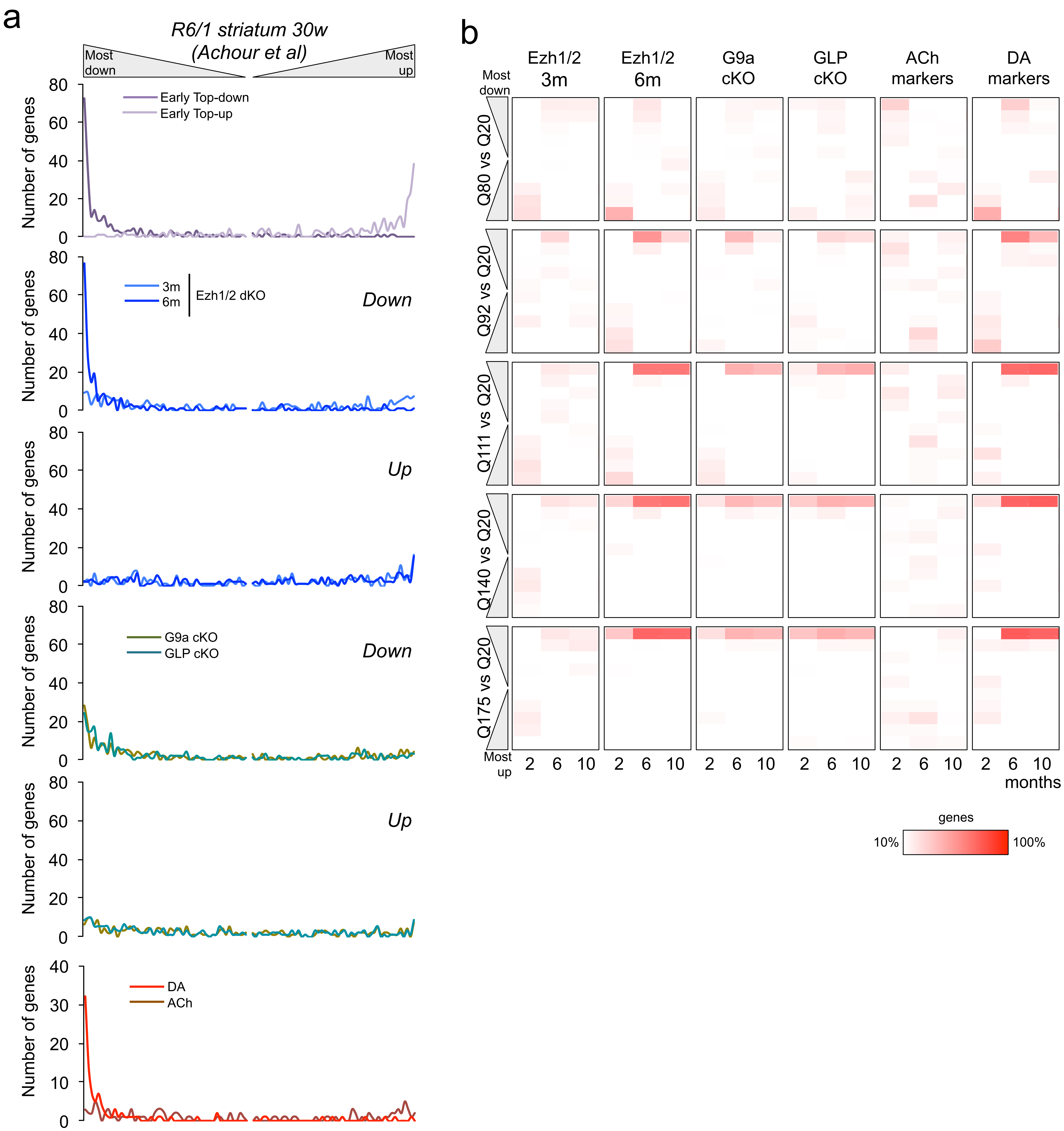

Supplementary Figure S4. CBP protein levels are slightly reduced in prodromic R6/1 brains

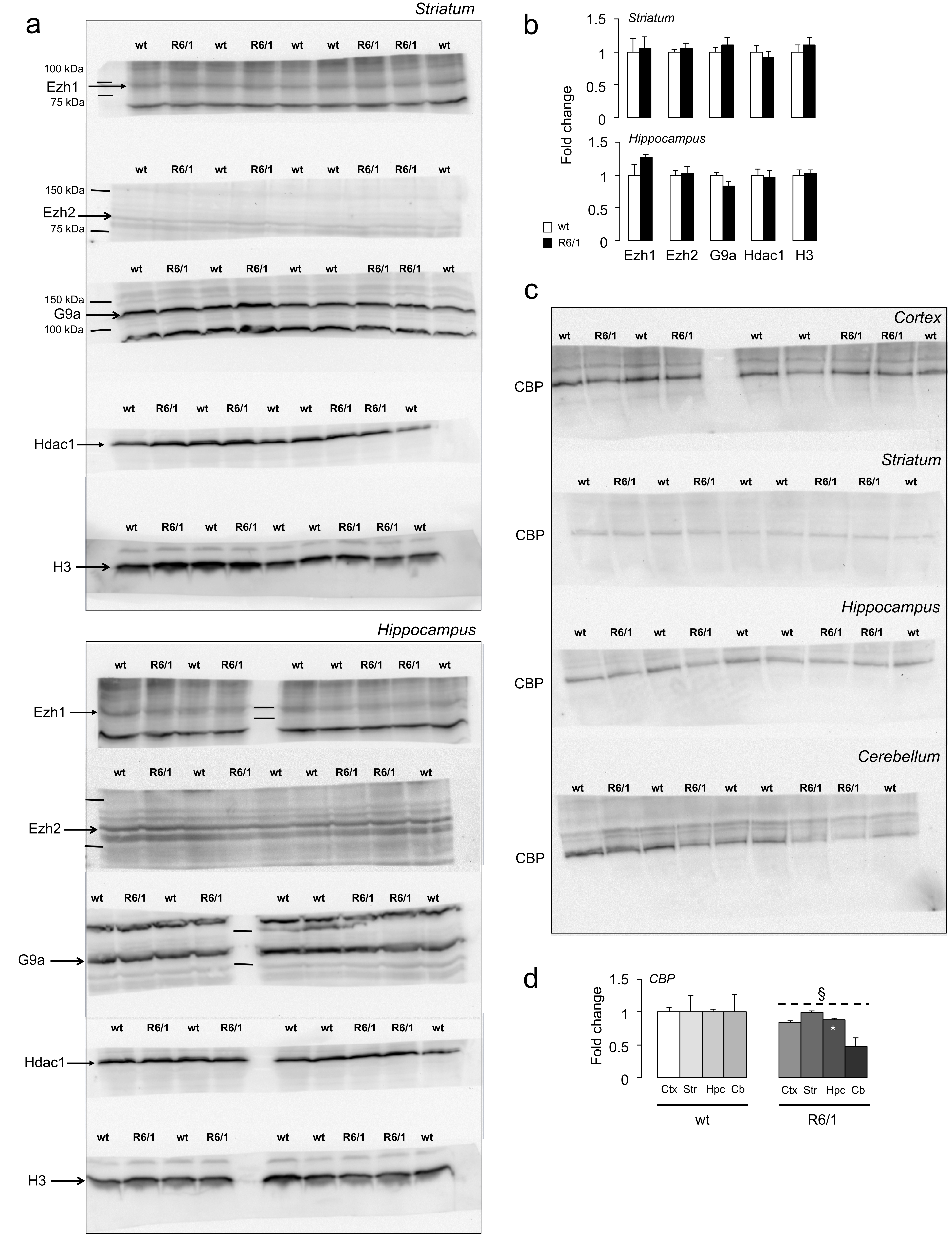

Supplementary Figure S5. Ablation of different histone methyltranferases leads to downregulation of striatal genes

a

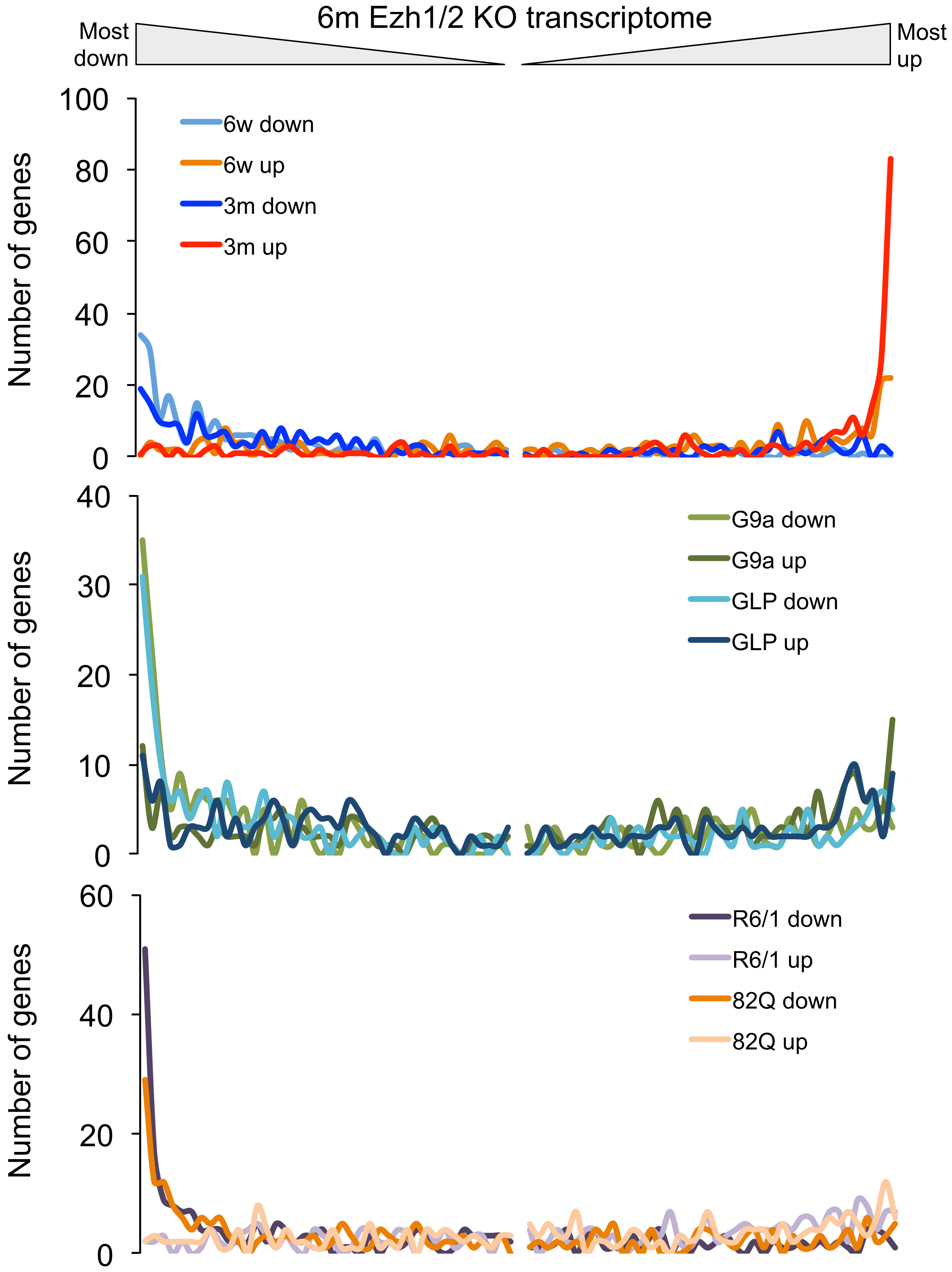

b

Ehmt2 (G9a) cKO	Ehmt1 (GLP) cKO	Ezh1 / Ezh2 dKO
Signal transduction	Signal transduction (GluR)	Synaptic transmission / Signal transduction
Cell differentiation / Morphogenesis	Cell development / Morphogenesis	Neuronal development
Ion transport		Ion transport (cation)
Metabolism (P, nucleobase)	Metabolism (P)	Metabolism (P)

c

Ehmt2 (G9a) cKO	Ehmt1 (GLP) cKO	Ezh1 / Ezh2 dKO
Ca^{2+} elevation	Ca^{2+} elevation	
Trp metabolism	Trp & melanin metabolism	
Organ & muscle development	Organ development	Organ development / Regionalization
Blood circulation / muscle contraction	Blood circulation / muscle contraction	
Hydrolase activity regulation		
		Transcription

Supplementary Figure S6. Anatomical markers of mouse developing brain in HD and epigenetic-related profiles

a

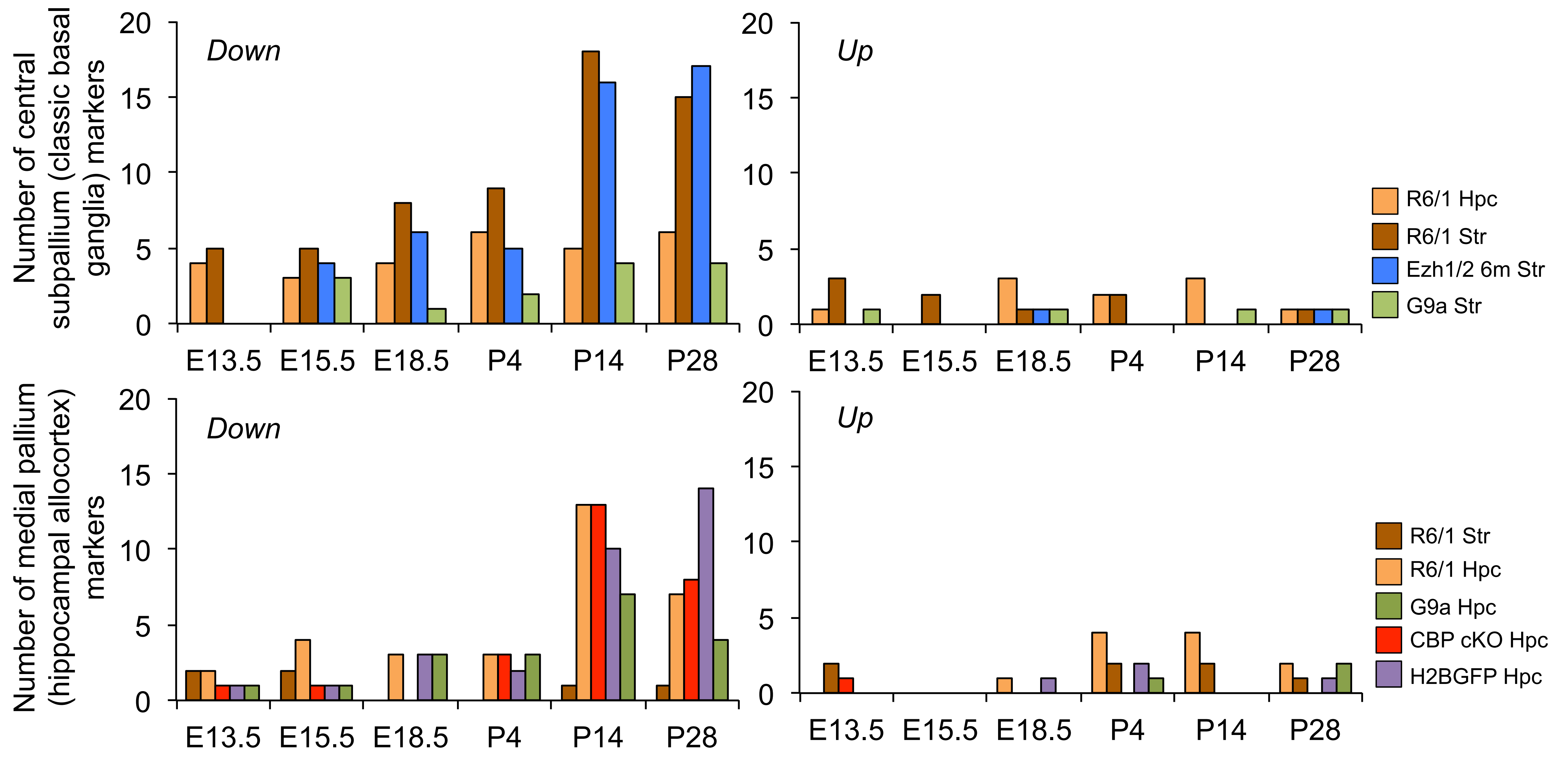

b

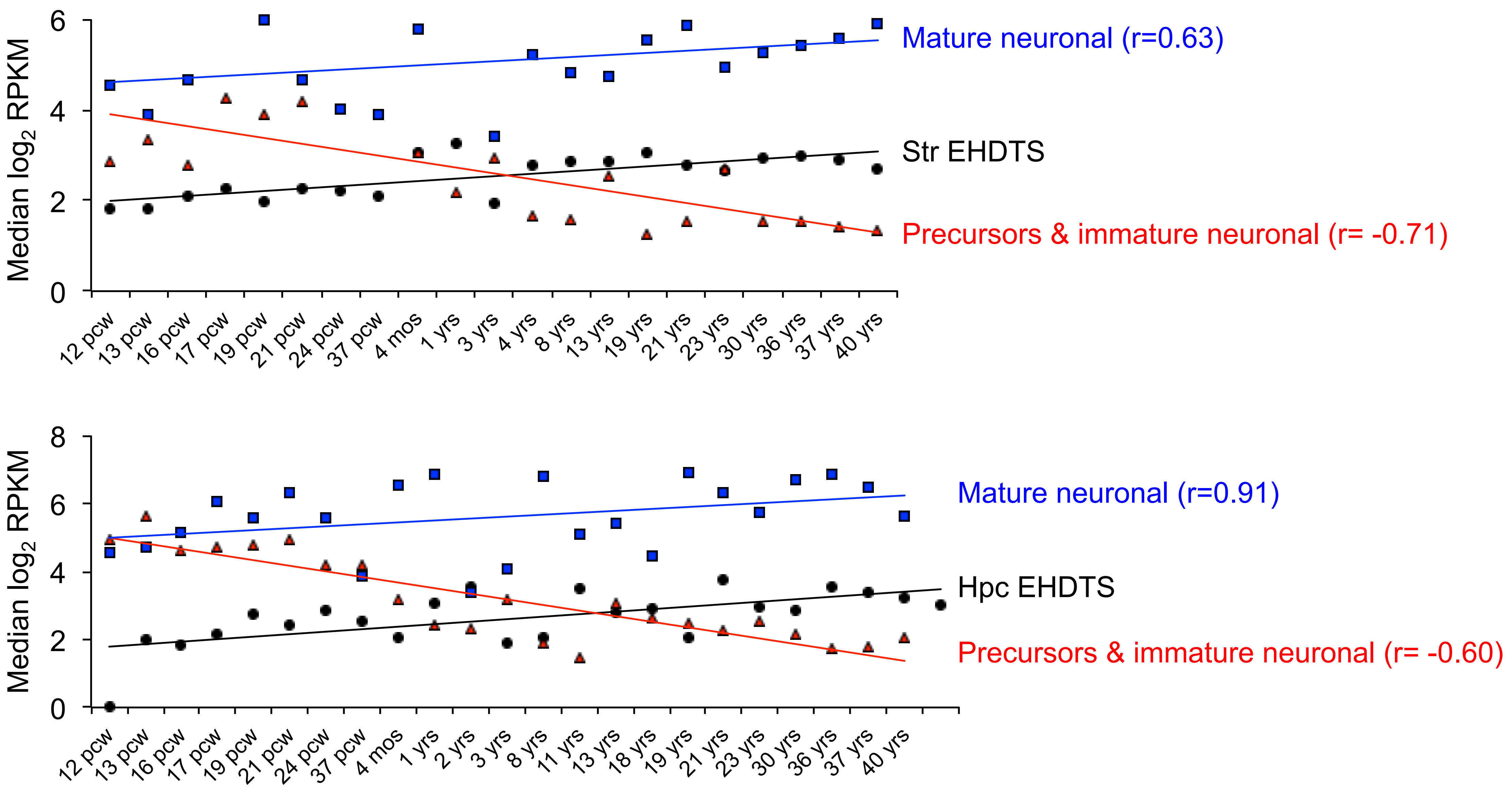

Supplementary Figure S7. The striatal early signatures contain genes with defective histone modifications

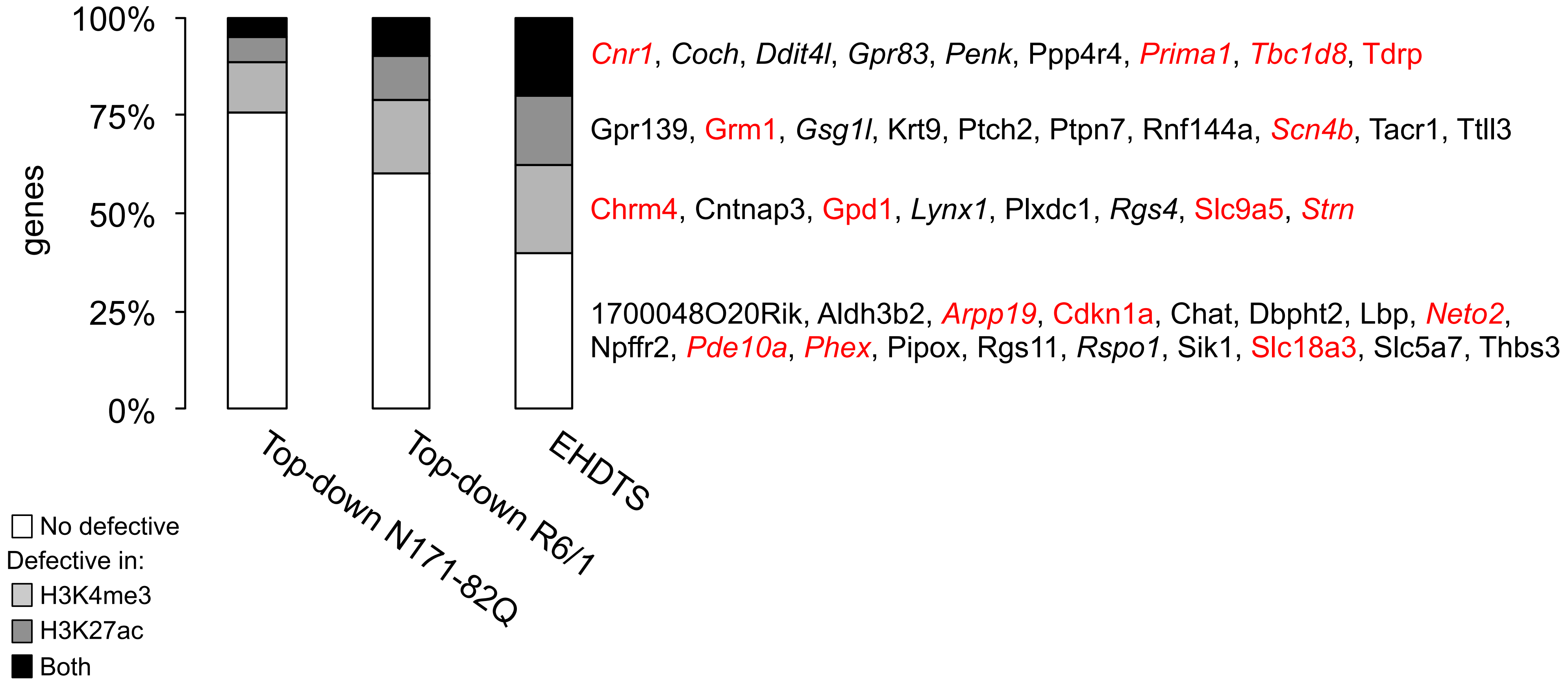

Supplementary Figure S8. Global epigenetic architecture is not altered in the striatum of R6/1 mice

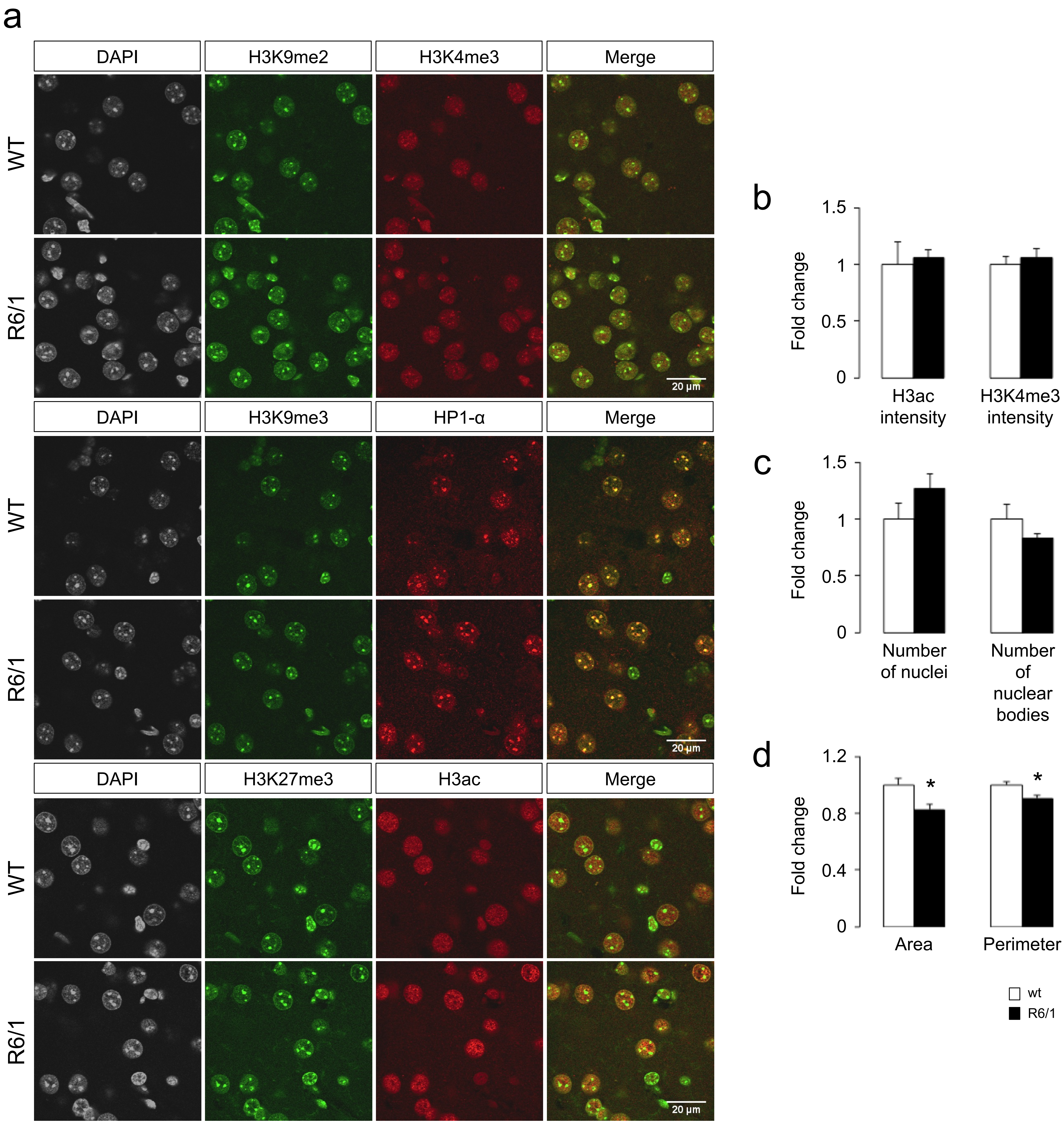

Supplement: Supplementary file 1 — Supplementary Figures S1-S8 [file 41598_2018_28185_MOESM1_ESM.pdf]
